# *Trans*-aconitic acid assimilation system as a widespread bacterial mechanism for environmental adaptation

**DOI:** 10.1093/ismejo/wrae198

**Published:** 2024-10-07

**Authors:** Cao Zheng, Dingqi Liu, Xinyu Lu, Huijun Wu, Jingyi Hua, Chuang Zhang, Kang Liu, Changchun Li, Jin He, Cuiying Du

**Affiliations:** Hubei Province Research Center of Engineering Technology for Utilization of Botanical Functional Ingredients, Hubei Key Laboratory of Quality Control of Characteristic Fruits and Vegetables, College of Life Science and Technology, Hubei Engineering University, Xiaogan, Hubei 432000, China; National Key Laboratory of Agricultural Microbiology, Hubei Hongshan Laboratory, College of Life Science and Technology, Huazhong Agricultural University, Wuhan, Hubei 430070, China; State Key Laboratory of Biocatalysis and Enzyme Engineering, Hubei Hongshan Laboratory, Hubei Collaborative Innovation Center for Green Transformation of Bio-Resources, Hubei Key Laboratory of Industrial Biotechnology, School of Life Sciences, Hubei University, Wuhan, Hubei 430062, China; Key Laboratory of Integrated Management of Crop Diseases and Pests (Ministry of Education), College of Plant Protection, Nanjing Agricultural University, Nanjing, Jiangsu 210095, China; Hubei Province Research Center of Engineering Technology for Utilization of Botanical Functional Ingredients, Hubei Key Laboratory of Quality Control of Characteristic Fruits and Vegetables, College of Life Science and Technology, Hubei Engineering University, Xiaogan, Hubei 432000, China; Hubei Province Research Center of Engineering Technology for Utilization of Botanical Functional Ingredients, Hubei Key Laboratory of Quality Control of Characteristic Fruits and Vegetables, College of Life Science and Technology, Hubei Engineering University, Xiaogan, Hubei 432000, China; Hubei Province Research Center of Engineering Technology for Utilization of Botanical Functional Ingredients, Hubei Key Laboratory of Quality Control of Characteristic Fruits and Vegetables, College of Life Science and Technology, Hubei Engineering University, Xiaogan, Hubei 432000, China; Hubei Province Research Center of Engineering Technology for Utilization of Botanical Functional Ingredients, Hubei Key Laboratory of Quality Control of Characteristic Fruits and Vegetables, College of Life Science and Technology, Hubei Engineering University, Xiaogan, Hubei 432000, China; National Key Laboratory of Agricultural Microbiology, Hubei Hongshan Laboratory, College of Life Science and Technology, Huazhong Agricultural University, Wuhan, Hubei 430070, China; Hubei Province Research Center of Engineering Technology for Utilization of Botanical Functional Ingredients, Hubei Key Laboratory of Quality Control of Characteristic Fruits and Vegetables, College of Life Science and Technology, Hubei Engineering University, Xiaogan, Hubei 432000, China

**Keywords:** microbial survival, metabolic adaptation, carbon source assimilation, bacterial inducible operon *tar*, *trans*-aconitic acid, aconitate isomerase TarA, *trans*-aconitic acid importer TarB, *Bacillus velezensis*

## Abstract

The ability of bacteria to use natural carbon sources greatly affects their growth and survival in the environment. Bacteria have evolved versatile abilities to use environmental carbon sources, but their diversity and assimilation pathways remain largely unexplored. *Trans*-aconitic acid (TAA), a geometric isomer of *cis*-aconitic acid involved in the tricarboxylic acid cycle, has long been considered a natural carbon source metabolizable by bacteria. However, its catabolism and ecological role in linking bacterial interactions with the environment remain unclear. Here, we identify a regulatory system in *Bacillus velezensis* FZB42 that is capable of sensing and catabolizing TAA. The system consists of a *tar* operon, an adjacent positive regulatory gene *tarR*, and a shared promoter. After receiving the TAA signal, the TarR protein interacts directly with the promoter, initiating the expression of the membrane transporter TarB and aconitate isomerase TarA encoded by the operon, which function in importing the TAA and isomerizing it into the central intermediate *cis*-aconitic acid. Subsequent soil colonization experiments reveal that TAA assimilating ability can give its coding bacteria a growth and competitive advantage. Bioinformatics analyses coupled with bacterial isolation experiments further show that the assimilation system of TAA is widely distributed in the bacterial domain, and its assimilating bacteria are also extensively distributed in nature, indicating an important role of TAA metabolism in bacterial carbon acquisition. This work emphasizes the importance of metabolic adaptation to environmental carbon sources for bacterial survival and may provide inspiration for engineering microbes with enhanced environmental competitiveness.

## Graphical Abstract

**Graphical Abstract:**
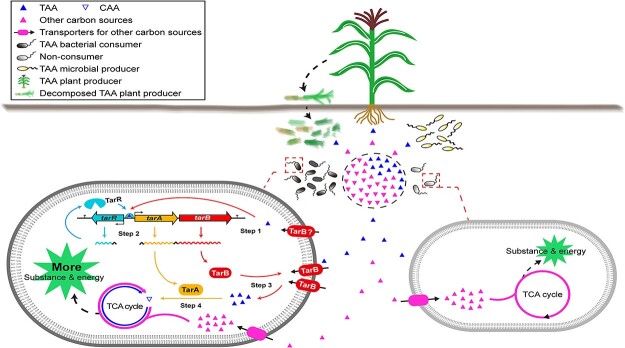

## Introduction

Carbon accounts for about half of the dry weight of microbial cells [1–3]; therefore, carbon source is the most needed nutrient and also an energy source, which is the key and prerequisite for the survival of microorganisms. The different abilities of microbes to utilize organic carbon sources determine their survival differences and ecological niches in the environment [4–7]. In the natural environment, low molecular weight sugars, organic acids, amino acids, and fatty acids are generally considered to be the most common components in the organic carbon pool [8], which are widely used by most microbial species. Their metabolic pathways have been well studied and have become classical biochemical pathways.

**Figure 1:**
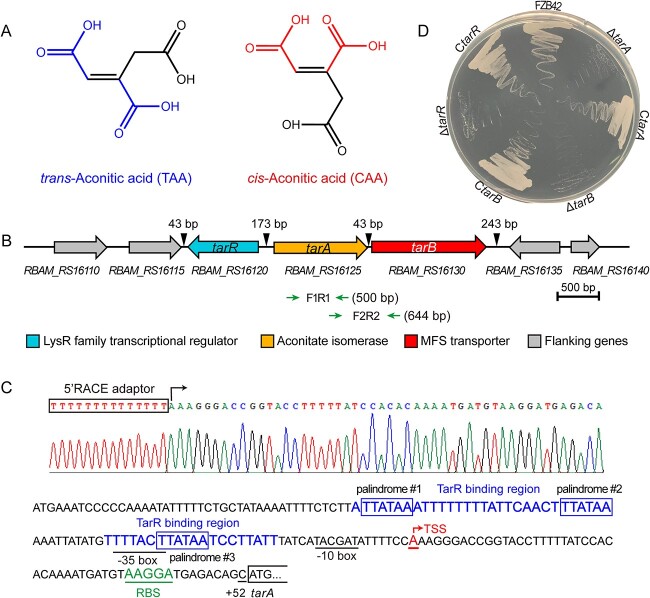
Genetic validation of the *B. velezensis* FZB42 TAA assimilation gene cluster. (A) Chemical structures of TAA and CAA geometric isomers. The configurational difference of the 1-carboxyl and the 6-carboxyl groups in TAA and CAA molecules on the opposite side and the same side of the double bond are highlighted. (B) Genetic organization of the *tar* genes in the FZB42 genome. Distances are shown to scale. Genes flanking the *tarR* and *tar* operon are: *RBAM_RS16110*, LacI family DNA-binding transcriptional regulator coding gene; *RBAM_RS16115*, ATP-dependent Clp endopeptidase proteolytic subunit ClpP coding gene; *RBAM_RS16135*, TIGR00730 family Rossman fold protein coding gene; and *RBAM_RS16140*, MazG-like family protein coding gene. Two pairs of *green* inverted arrows indicate the amplified regions in the *tar* operon analysis, and the sizes of amplified products F1R1 and F2R2 are given. (C) Structure diagram of the *tar* promoter. (D) TAA utilization phenotypes of FZB42, *tar* gene mutants, and their corresponding complementary strains on ACO medium plates.

In addition to these traditional carbon sources, there are likely many other important components that play a role in driving the exchange between microorganisms and the environment. For example, the natural product *trans*-aconitic acid (TAA) [9] (Fig. 1A), the geometric isomer of *cis*-aconitic acid (CAA) in the tricarboxylic acid (TCA) cycle, is one such under-appreciated carbon source. In terms of molecular property of TAA, due to its structural similarity to CAA, TAA can competitively inhibit aconitase [10], an enzyme that uses CAA as both a product and a substrate in the TCA cycle. However, in nature, TAA can be synthesized and secreted by plants as components of root exudates [11, 12]. For example, in barley (*Hordeum leporinum*), reed grass (*Phalaris tuberose*), and western larkspur (*Delphinium hesperium*), TAA concentrations reach astonishing levels of 3.5%, 4.2%, and 12.2% of dry weight, respectively [13, 14]; in maize, TAA accounts for 95% of total aconitic acid and often reaches milligram of fresh weight [15]. Bacteria also synthesize TAA. For example, in the nematode pathogen *Bacillus thuringiensis*, TAA can be synthesized as one of the main nematicidal toxins and accumulated extracellularly [9].

From 1961 to the present, research on TAA has focused almost exclusively on revealing its remarkable natural occurrence and biological effects, such as killing of plant-parasitic nematodes [9], protection against brown planthopper feeding [16, 17], inhibition of growth and transformation of *Leishmania* parasites [18], and anti-inflammatory effects on various mammalian diseases [19]. However, as a naturally occurring high-yield molecule, TAA was known to be metabolized by bacteria as early as 1961 [20], but unfortunately, few studies have explored how TAA is metabolized, whether this ability is widespread in bacteria, and whether it is active in mediating interactions with the environment and microbiota.

*B. velezensis* FZB42 has become a model bacterium for the study of microbe-environment interaction and is also a successful biofertilizer and antimicrobial strain used in agriculture [21, 22]. In this work, we found that FZB42 has a strong and efficient TAA assimilation ability (Fig. S1A) and encodes a metabolic regulatory system that can sense and assimilate TAA. The system consists of a TAA assimilation-related (*tar*) operon and a regulatory gene *tarR*. The TarR protein receives the TAA signal and then activates the expression of the *tar* operon, which includes aconitate isomerase TarA (a core enzyme that converts TAA) and a TAA importer protein TarB. Further, the TAA assimilation system is found to provide significant growth and competitive advantages to host bacteria when colonized in soil and is active and widespread in the bacterial domain and natural ecosystems. Therefore, this study reveals the role of TAA molecules as important carbon sources in nature and the role of their catabolism in bacterial carbon acquisition and microbe-environment crosstalk, which will provide new perspectives for understanding the metabolic adaptations of microorganisms and designing tailored interactions between specific microbes and target environments.

## Materials and methods

### Bacterial strains, plasmids, and culture conditions

The bacterial strains and plasmids used in this study are listed in Table S1. For general cell propagation, *Escherichia coli* and *B. velezensis* strains were cultured in lysogeny broth (LB) medium at 37°C. For the TAA assimilation ability test, strains were collected in the logarithmic growth phase to prevent spore formation and false-negative results caused by dormancy, washed with ultrapure water to remove residual nutrients, and then inoculated into minimal ACO medium (7.5 g/L TAA, 2 g/L (NH_4_)_2_SO_4_, 1 g/L K_2_HPO_4_, 0.5 g/L MgSO_4_, and 0.1 g/L FeCl_3_·6H_2_O, pH 7.0) [23], or modified ACO medium (containing 1 g/L glucose and 2 g/L TAA). Antibiotics were added at appropriate final concentrations: 100 μg/ml ampicillin, 25 μg/ml erythromycin, 30 μg/ml (for *E. coli*) and 5 μg/ml (for *B. velezensis*) chloramphenicol, 50 μg/ml (for *E. coli*) and 10 μg/ml (for *B. velezensis*) kanamycin.

### Gene deletion and complementation in *B. velezensis* FZB42

To construct the *tar* gene deletion and complementation vectors, *Cm* and *Kan* resistance cassettes (Tables S1 and S2) were used to construct the corresponding pΔ*tar* and pC*tar* vectors based on the pMD19-T vector, respectively. In the gene complementation, the α-amylase gene locus (*amyE*, *RBAM_RS01650*) [24] of the FZB42 genome was used to integrate the intact *tar* gene. Transformation of *B. velezensis* was conducted as previously described [24]. Generally, *B. velezensis* cells were cultured in 20 ml of SPI medium (0.19% (NH_4_)_2_SO_4_, 1.36% K_2_HPO_4_·3H_2_O, 0.58% KH_2_PO_4_, 0.10% Trisodium citrate dihydrate, 202 μl 5% MgSO_4_·7H_2_O, 202 μl 50% w/v glucose, and 202 μl 1% w/v CAYE) at 37°C, 200 rpm, to an OD_600_ value of 1.4–1.5, and 2.5 ml of the culture was then inoculated into 20 ml of SPII medium (19.60 ml SPI, 200 μl 50 mM CaCl_2_, and 200 μl 250 mM MgCl_2_). After culturing at 37°C and 100 rpm for 1.5 h, cells were induced with 250 μl of 10 mM EGTA for 10 min to make the cells competent. The linearized vector of pΔ*tar* or pC*tar* digested with *Bam*HI was added and cultured at 37°C and 100 rpm for 45 min. Then 800 μl of LB medium was added and cultured at 37°C and 200 rpm for 2 h. The cells were then spread on resistant plates for transformant screening.

### RNA manipulation for TarR regulatory assays

Total RNA of *Bacillus* strains cultured in modified LB liquid medium (5 g/L peptone, 5 g/L NaCl, 2.5 g/L yeast extract, with or without 7.5 g/L TAA, pH 7.0) for 20 h was extracted, digested, and reversely transcribed as previously described [25]. For reverse transcription PCR (RT-PCR) and quantitative real-time PCR (qRT-PCR) assays, the internal reference gene *23S rRNA* (Table S2) and the 2^-ΔΔCt^ method were used. To determine the transcription start site (TSS), a terminal deoxynucleotidyl transferase-based 5′-RACE experiment was conducted as previously described [25].

### Expression and purification of TarA, TarB, and TarR proteins

For purification of TarB membrane protein, a transformed *E. coli* C43(DE3) strain was induced with isopropyl-β-D-thiogalactoside (IPTG) at a final concentration of 0.5 mM, harvested after 8 h of growth at 37°C, and resuspended in buffer A (25 mM Tris-HCl, 150 mM NaCl, and 10 mM imidazole, pH 7.6). After high-pressure homogenization, the supernatant was obtained by centrifugation at 34 570 *g* for 1 h at 4°C. The precipitate was collected by ultracentrifugation (Beckman Coulter Optimal XE-100, USA) at 200 000 rpm for 1 h, dissolved in buffer A (1% w/v *n*-dodecyl β-D-maltoside), stirred for 5 h, and centrifuged at 34 570 *g* to obtain the supernatant containing TarB, which was then purified by Ni-NTA affinity [26], quickly concentrated by ultrafiltration (10 kDa), and further purified by size exclusion chromatography using a Superdex 200 Increase 10/300 GL column. Except for TarB, all other proteins prepared in this work were induced in *E. coli* Rosetta(DE3) with 0.2 mM IPTG (final concentration) at 16°C overnight and purified by Ni-NTA affinity column.

For Western blot assays, cell lysis and centrifugation methods were similar to those described above. The supernatant and pellet collected after ultracentrifugation at 200 000 rpm (Beckman Coulter Optimal XE-100, USA) for 1 h were samples of the cytoplasm fraction and membrane fraction, respectively.

### Enzymatic assays of AI activity

Chemical standards of CAA and TAA (purity >98%) were purchased from Aladdin (Shanghai, China) and Tokyo Chemical Industry (Japan), respectively. During the preparation of the reaction system, the final concentration of each test enzyme was adjusted and normalized to 1 μM. When TarA activity was set to 100%, the relative AI activities of 16 TarA homologs were calculated based on the ratio of the peak area of product formed by TarA homologues to the peak area of product formed by TarA.

### Fluorescence microscopy

The coding sequences (CDSs) of *tarB* and *gfp* digested from plasmid pAD43-25 at the *Xba*I and *Hin*dIII sites and connected by a 10-aa peptide linker coding sequence were spliced by overlap extension PCR (SOE-PCR), inserted into pAD43-25, and transformed into FZB42 to generate the recombinant FZB42-TarB-GFP strain. The strain FZB42-GFP containing the empty pAD43-25 plasmid was used as a control. Cell preparation and image processing were conducted using a Nikon structured illumination super-resolution microscope (N-SIM; Nikon Corporation, Japan) as previously described [9].

### Assays of *B. velezensis* FZB42 and Δ*tarB* in modified ACO liquid medium

FZB42 and Δ*tarB* cultures were adjusted to a final OD_600_ value of 0.01, inoculated into modified ACO liquid medium, and grown at 37°C for 48 h. Samples were taken every 2 h, and the OD_600_ value of cell growth was determined using a microplate reader, and the residual levels of glucose and TAA in the bacterial supernatants were determined using a Glucose Content Assay kit (Biosharp, China) and as previously described [9], respectively. Meanwhile, samples were taken at 10 h, washed three times with ultrapure water, ground with liquid nitrogen, and then dissolved in 1 ml ultrapure water. After centrifugation at 17 220 *g* for 15 min, the supernatant was used for LC-Q-TOF-MS detection of intracellular TAA as previously described [9].

### Microscale thermophoresis (MST)

The procedure was done according to the instructions of the Monolith His-Tag labeling kit (NanoTemper Technologies, Germany). Generally, TarB or TarR protein (800 nM) and dye (100 nM) were mixed evenly, incubated for 30 min, and then centrifuged to obtain the supernatant. One micromolar TAA ligand was set as the highest concentration, followed by the preparation of 16 gradient concentrations. Finally, 10 μl of labeled protein was added to each tube and loaded onto a model with a standard capillary. The scanning parameters are MST Power: 40% and LED Power: 80%. Fluorescence was measured using a Monolith NT.115 and data analyzed using MO. Affinity Analysis V2.3^*^ (NanoTemper Technologies, Germany).

### Electrophoretic mobility-shift assay (EMSA)

The promoter DNA was fluorescently labeled (Table S2) and purified using a Nucleic Acid Purification kit (Axygen, USA). The working concentrations of DNA probes and TarR protein are detailed in the corresponding figures. To determine the role of the “TTATAA” sequence in the *P_tar_*-TarR interaction, “TTATAA” was mutated to “CCGCGG,” and the *P_tar_* DNA mutants were de novo synthesized (GenScript Biotech Corporation, China). The procedures for EMSA were conducted as previously described [27].

### DNA foot-printing assay

In a 200-μl reaction system, 1 000 ng of 5′-FAM-labeled *P_tar_* DNA and 5 μM TarR (final concentration) was mixed (in the control experiment, bovine serum albumin was used instead of TarR) in 10 mM Tris-HCl (pH 7.8) with 10 mM MgCl_2_, 1 mM CaCl_2_, 0.4 mM dithiothreitol, 100 mM KCl, and 5% glycerol, and incubated at room temperature. After 30 min, 0.5 U of RNase-free DNase I (Roche, Basel, Switzerland) was added for digestion at 25°C for 3 min. The reaction termination, precipitation, and analysis procedures were done as previously described [28].

### Growth assays in soil

Soil was collected from the vegetable field of Hubei Engineering University (30°94′N and 113°91′E), subjected to physical and chemical characterization (Table S3), thoroughly crushed and passed through a 40-mesh sieve, and finally sterilized at 121°C for 1 h. Twenty grams of sterile soil was inoculated in a sterile Petri dish, and 10^7^ cfu/g of bacterial suspension (FZB42 or Δ*tarA* vegetative cells, cultured at 37°C for 9 h, washed and resuspended in sterile ultrapure water, adjusted to the same cell turbidity). Then, 0 (control group) and 100 mg/kg (test group) of TAA aqueous solution (pH 7.0) were added to the soil. The soil was further cultured at 25°C and 40% relative humidity. One gram of soil was sampled from each treatment every 1–5 days and shaken in 9 ml of sterile ultrapure water at 25°C and 200 rpm for 20 min. The supernatant was diluted 10-fold in a gradient, spread on LB plates, incubated at 30°C overnight, and then counted.

### Competition assays in soil

FZB42 or Δ*tarA* cultures were adjusted to a cell density of 10^7^ cfu/ml using turbidimetry, then mixed in equal volumes and inoculated into 20 g of sterile soil pre-mixed with 100 mg/kg of TAA (test group) and another 20 g of sterile soil without TAA addition (control group). The procedures for culturing and sample preparation were described above. Δ*tarA* colonies were picked using *Cm^r^* selective plates (5 μg/ml) and counted as previously described [29].

### Phylogenetic analysis of TAA assimilation genes in bacterial taxa

For genomic data acquisition, a total of 16 986 complete bacterial genomes were obtained from the RefSeq database (updated to March 2023) of NCBI (National Center for Biotechnology Information).

To search for homologous proteins of TarA, TarB, and TarR in bacterial genomes, we used the hmmsearch tool of the HMMER (Version 3.3.2) software package to create an hmm seed file. The specific operations are as follows: Taking TarA, TarB, and TarR protein sequences as query sequences, blastp (Version 2.13.0+) was used to search for homologous proteins in the NCBI database, and the 100 sequences with the highest scores were selected as original sequences. These sequences were then aligned using MAFFT software (Version 7.505), and the hmmbuild tool was used to create an hmm file. Next, blastp was used to align the potential Tar proteins screened from the genomes with the corresponding TarA, TarB, and TarR protein sequences, and proteins with a similarity >30% were defined as Tar homologous proteins.

To construct the phylogenetic tree of TarA and its homologous proteins, all TarA homologous proteins were first clustered at 50% sequence identity using the CD-HIT tool (Version 4.8.1). Then, the protein sequences were aligned using MAFFT software, and the maximum likelihood trees were constructed using Fasttree (Version 2.1.11). Finally, the phylogenetic trees were modified using the ggtree package (Version 3.8.2) in the R language (Version 4.3.1).

### Isolation and identification of TAA-assimilating environmental bacteria

A total of 32 environmental samples were collected from Xiaogan city (32°92′N and 113°91′E) and Wuhan city (30°48′N and 114°37′E) in Hubei Province, China (Table S4). One gram or 1 ml of environmental sample was inoculated into 20 ml of LB liquid medium. After vigorous growth at 30°C for 2 h, 1 ml of bacterial supernatant was spread on ACO plates and grown at 30°C for 24 h. Colonies were picked and sent to Tsingke Biotechnology Co., Ltd (China) for species identification.

### Identification of AI-encoding genes in TAA-assimilating bacteria

Twelve out of sixty-six bacterial isolates representing different living environments, including plant-associated (beneficial and pathogenic), animal-associated (pathogenic), and free-living (neutral), were selected for whole-genome sequencing by Bioyi Biotechnology Co., Ltd (China). Each genome sequence of the 12 strains was aligned with the TarA protein sequence using the tblastn tool to identify AI homologous genes.

### Statistical analysis

All our experiments were conducted in three biological replicates and three technical replicates for each treatment, and values are expressed as mean ± standard deviation. One-way ANOVA was conducted using Tukey’s honest significant difference test with an error probability of *P* < .01 (^**^) and *P* < .0001 (^****^) to determine statistically significant differences in comparison of AI protein activities and in *tar* gene expression levels in the TarR regulation studies. IBM SPSS (Statistical Package for the Social Sciences) software (Version 20.0) was used for these analyses.

## Results

### *tarA* and *tarB* genes constitute an operon and are responsible for TAA assimilation in *B. velezensis* FZB42

AI was proposed as a new enzyme as early as 1961 [20] and was considered to be responsible for TAA utilization in bacteria. In 2017, we identified an AI-encoding gene, *tbrA* (TAA biosynthesis-related gene A); however, it mediates TAA biosynthesis in *B. thuringiensis* [9]. Although *tbrA* mediates reversely, it is the only AI sequence available to date. Therefore, we used the TbrA protein sequence to do tblastn analysis against the FZB42 genome, and fortunately found the only homolog of this gene, *RBAM_RS16125* (1 131 bp, 376 aa) (Fig. 1B). Its protein sequence shares 33% identity and 94% coverage with *B. thuringiensis* TbrA and belongs to the same PrpF superfamily as TbrA after conserved domain (CD) search (Fig. S1B). Based on these findings, we proposed that *RBAM_RS16125* is an AI-encoding gene with the potential to isomerize TAA in *B. velezensis* and therefore named it TAA assimilation-related gene A (*tarA*). Meanwhile, we noticed that the gene *RBAM_RS16130* (1 377 bp, 458 aa) located 43 bp downstream of *tarA* (Fig. 1B) formed an operon with *tarA* (Fig. S1C) and determined that the TSS of the operon *tar* was located 52 bp upstream of the start codon of the *tarA* CDS (Fig. 1C). CD search further reveals that *RBAM_RS16130* encodes a major facilitator superfamily (MFS) transporter with 12 transmembrane-helices (Fig. S1D). Considering the domain and function annotations, as well as the similar gene organization to the TAA biosynthesis operon *tbr* in *B. thuringiensis* (containing the AI-encoding gene *tbrA* and the TAA exporter-encoding gene *tbrB*), we proposed that *RBAM_RS16130* was associated with the intracellular transport of TAA in TAA assimilation and named it *tarB*.

To test whether the *tar* operon is responsible for TAA assimilation in FZB42, we tested the growth of single gene deletion mutants Δ*tarA* and Δ*tarB* separately and found that although they grew normal in liquid LB medium like FZB42 (Fig. S2A), both mutants could not grow on solid (Fig. 1D) or liquid ACO media (Fig. S2B). However, complementation of *tarA* or *tarB* restored TAA assimilation ability (Fig. 1D).

These results genetically confirmed that the operon composed of *tarA* and *tarB* is responsible for the assimilation of the TAA carbon source in *B. velezensis* FZB42.

### TarA is an AI in *B. velezensis* that converts TAA to CAA

**Figure 2:**
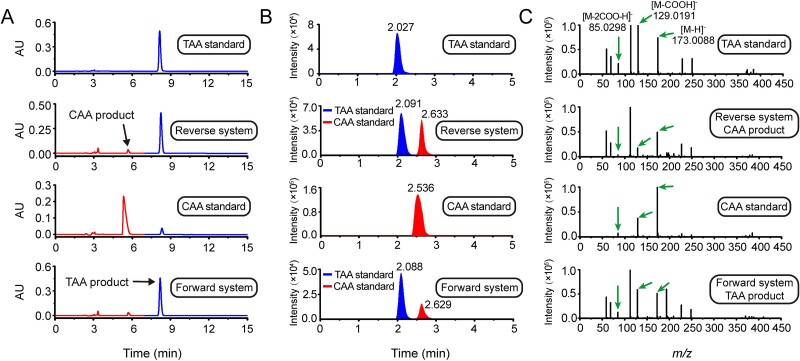
AI activity of the TarA protein. (A) HPLC analysis of AI activity of TarA in an *in vitro* catalytic system. (B, C) LC-Q-TOF-MS verification of CAA formation in a reverse system and TAA formation in a forward system, respectively. The values next to the chromatographic peaks indicate the retention times. The *m/z* 173.0088 ion is the mass of the [M-H]^−^ ion of aconitic acid. The signals at *m/z* 129.0191 and 85.0298 are the decarboxylation products of one and two carboxyl groups from the [M-H]^−^ ion, respectively.

To confirm the AI activity of the TarA protein, the interconversion activity of the recombinant TarA-His_6_ protein was tested *in vitro* using TAA and CAA as substrates. Distinct formations of CAA product with TAA substrate and TAA product with CAA substrate were detected by both HPLC and LC-Q-TOF-MS (Fig. 2), demonstrating that TarA catalyzes the reversible reaction from TAA to CAA, but is much more efficient in forming TAA. These properties are consistent with the classic characteristics of AI enzymes. We further determined the effects of pH, ionic strength, and various metal ions on TarA activity, as well as the *K*_m_, *v*_max_, *k*_cat_, and *k*_cat_*/K*_m_ kinetic constants of the TarA isomerization reaction to further elucidate its catalytic characteristics (Fig. S3). The constants measured via Lineweaver–Burk plots (Fig. S3E and F) are listed in Table S5, quantitatively confirming the equilibrium preference of TarA in TAA formation.

These results demonstrated that *B. velezensis* TarA is an AI that can isomerize TAA to CAA.

### TarB is a membrane importer of TAA in *B. velezensis*

**Figure 3:**
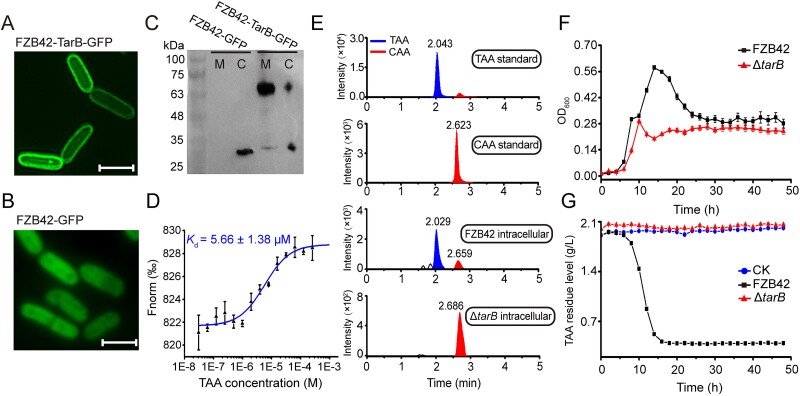
Characterization of TarB as a TAA membrane importer. (A) Fluorescence micrograph of TarB-GFP fusion protein in the FZB42-TarB-GFP strain, and (B) GFP protein in the FZB42-GFP strain. Scale bar, 2 μm. (C) Western-blot confirmed the presence of TarB in the cell membrane fraction. M: membrane fraction, C: cytoplasmic fraction. (D) MST revealed the binding effect and constant between TarB protein and TAA substrate. (E) LC-Q-TOF-MS analysis of the presence of TAA in the intracellular fractions of FZB42 and Δ*tarB* strains. Both cells were examined at the 10 h time point of the growth phases in Fig. 3F. The ion at *m*/*z* 173.0088, represents the mass of aconitic acid in [M-H]^−^ mode and was used to extract CAA and TAA signals from the total ion chromatogram (TIC). The retention times of the extracted CAA and TAA signals are provided. (F) Growth curves and (G) TAA residual levels in the supernatants of FZB42 and Δ*tarB* strains cultured in modified ACO liquid medium.

TarB is predicted as a MFS transporter with 12 transmembrane helices via bioinformatics. To verify its function as a cell membrane importer of TAA, the membrane subcellular localization of the TarB protein was first determined. Through a microscope, we observed a strong green fluorescent signal on the cell membrane of the FZB42-TarB-GFP strain, indicating the localization of TarB-GFP fusion protein on the membrane. In addition, only a weak green signal was observed in the cytoplasm due to the dynamic balance of production and loss of TarB-GFP protein (Fig. 3A). In contrast, as a control protein, GFP was only uniformly distributed in the cytoplasm (Fig. 3B). Western blot confirmed the presence of TarB-GFP in cell membrane extract (Fig. 3C). A molecular binding assay of purified TarB protein and TAA substrate by MST further reveals significant ligand dose-dependent changes in fluorescence intensity (*K*_d_ = 5.66 ± 1.38 μM) (Fig. 3D), demonstrating that TarB, a *B. velezensis* membrane protein, binds to the TAA molecule with moderate strength.

Then, the intracellular contents of FZB42 and Δ*tarB* strains cultured in modified ACO liquid medium were analyzed by LC-Q-TOF-MS. TAA was only detected in FZB42 cells but not in Δ*tarB* cells (Fig. 3E), suggesting that TAA is unable to enter the cells when *tarB* is deleted. From 0 to 6 h of the culture (Fig. 3F), FZB42 and Δ*tarB* appear to consume only glucose and not TAA (Fig. S4 and Fig. 3G). From 6 to 10 h, Δ*tarB* continues to consume glucose that is depleted at 10 h (Fig. S4). However, starting at 6 h, FZB42 used TAA and glucose as carbon sources, which are depleted up at 14 h (Fig. 3G) and 10 h (Fig. S4), respectively. In contrast, Δ*tarB* shows a complete impairment in the use of TAA, as Δ*tarB* is unable to transport TAA into the cells throughout the culture period (Fig. 3G). Therefore, when the only available carbon source, glucose, was depleted, Δ*tarB* reaches its maximum OD_600_ value at 10 h (Fig. 3F); whereas FZB42 underwent a diauxic growth within 10–14 h and reaches a population twice larger than that of Δ*tarB* (Fig. 3F), further confirming the TAA-importing function of TarB.

Taken together, these results demonstrate that TarB is a membrane importer of TAA molecules in *B. velezensis*.

### TAA signaling activated positive control of TAA assimilation by TarR in *B. velezensis*

**Figure 4:**
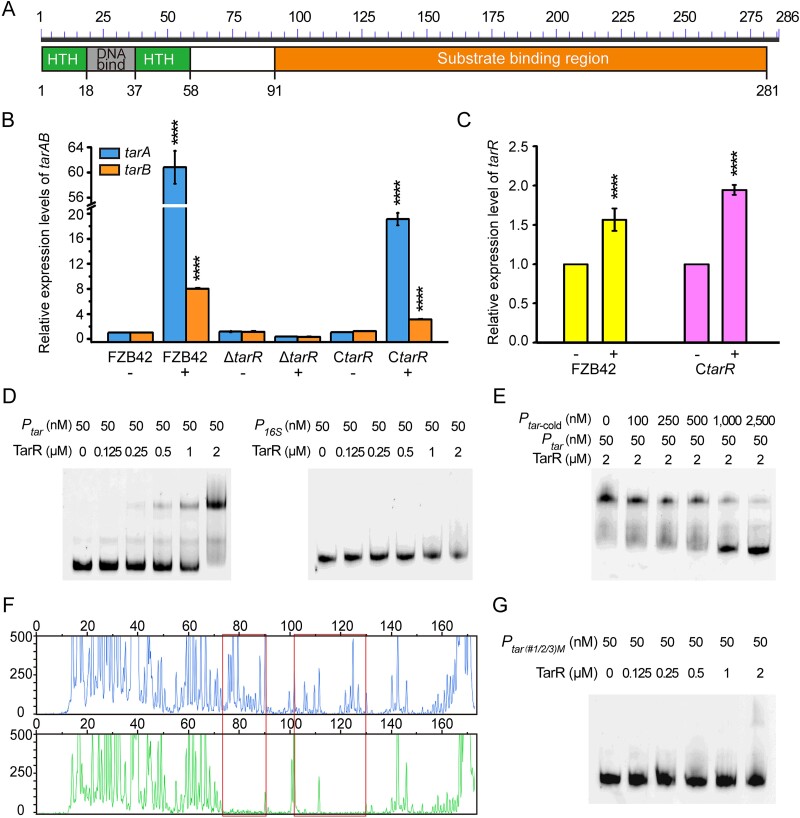
Regulatory properties of TarR. (A) Secondary structure analysis of TarR using the PROSITE tool of the Expasy database. The helix-turn-helix (HTH) motif and DNA-binding site at the N-terminus, and substrate binding region at the C-terminus are indicated. (B) Relative expression of *tarA* and *tarB* genes in FZB42, *ΔtarR,* and *CtarR* strains, and (C) the *tarR* gene in FZB42 and *CtarR* strains, with (+) and without (−) TAA induction. The transcription level of *tar* genes in FZB42 without TAA induction was defined as 1. The *23S rRNA* gene was used as a reference. (D, E) *In vitro* EMSA assays to determine the specific binding of TarR to the *P_tar_* DNA region. (F) DNase I foot-printing assay of the protected region of the *tarA* promoter by TarR (*red* boxes). (G) EMSA detection of the interaction between TarR and mutant *P_tar_* DNA, *P_tar(#1/2/3)M_*, in which the three palindromic sequences of “TTATAA” were changed to “CCGCGG”.

We noticed that one gene, *RBAM_RS16120* (861 bp), annotated as encoding a LysR-type transcriptional regulator (LTTR), is located 173 bp upstream of the AI gene *tarA* and is transcribed in an opposite direction with *tarA* (Fig. 1B). Further secondary structure analysis of the *RBAM_RS16120* gene product shows that the amino terminus (1–58 aa) contains a helix-turn-helix (HTH) DNA binding motif, and the carboxyl terminus (91–281 aa) contains a substrate binding region connected to the HTH motif via a 32-aa hinge region (59–90 aa) (Fig. 4A). This structure exhibits the typical domain organization of bacterial LTTRs [30]. Considering the functional annotation and the sharing of the promoter region with *tarA*, the *RBAM_RS16120* gene was considered to have a potential regulatory function on the TAA assimilation ability in *B. velezensis* and was therefore named *tarR*.

To test the regulatory function of *tarR* on TAA assimilation, we compared the growth of the Δ*tarR* mutant with that of the starting strain FZB42. Although showing a normal growth in LB medium like FZB42, Δ*tarR* is unable to grow on ACO medium (Fig. 1D and Fig. S2), implying positive regulation of TAA assimilation by *tarR*. Then, qRT-PCR analysis shows that in FZB42, when TAA was added, the transcription of *tarA* and *tarB* is significantly upregulated (Fig. 4B), confirming that the function of TAA assimilation in *B. velezensis* requires TAA molecules for activation. However, when *tarR* is deleted in FZB42, the expression of the *tar* operon cannot be activated despite the presence of TAA (Fig. 4B). Upon complementation with *tarR* in C*tarR*, transcription of *tarA* and *tarB* restores normal response to the TAA molecule (Fig. 4B). These results confirm the positive regulation of the inducible expression of *tar* by TarR and suggest a role for TarR in the reception of TAA signals. Moreover, *tarR* expression also responds positively to TAA induction (Fig. 4C). Next, to determine whether TarR functions as a direct regulator of the *tar* operon, EMSAs were conducted on the TarR protein with *tar* promoter region (*P_tar_*), and the *16S rRNA* promoter (*P_16S_*) region as a control. Specific interactions appear in the TarR-*P_tar_* system but not in the control (Fig. 4D). In the competition assay, when fixed amounts of TarR and labeled probe were incubated with increasing amounts of non-labeled cold probe, the shifted bands gradually disappeared (Fig. 4E), indicating that the cold probe competitively binds TarR, resulting in reduced amounts of labeled probe-TarR complex. These results show that TarR recognizes and binds *P_tar_* specifically. To further define the binding site(s) of TarR in *P_tar_*, two independent protected regions are identified by DNase I footprinting assay (Fig. 4F and Fig. 1C), showing a dual-site interaction between TarR and the *tar* promoter. Within the two regions, three 6-bp palindromic motifs “TTATAA” were identified. Mutation of one or three motifs caused the shifted bands to weaken or even disappear (Fig. S5 and Fig. 4G), demonstrating that “TTATAA” is a key sequence mediating *P_tar_*-TarR interaction and that all three palindrome sequences are essential.

**Figure 5:**
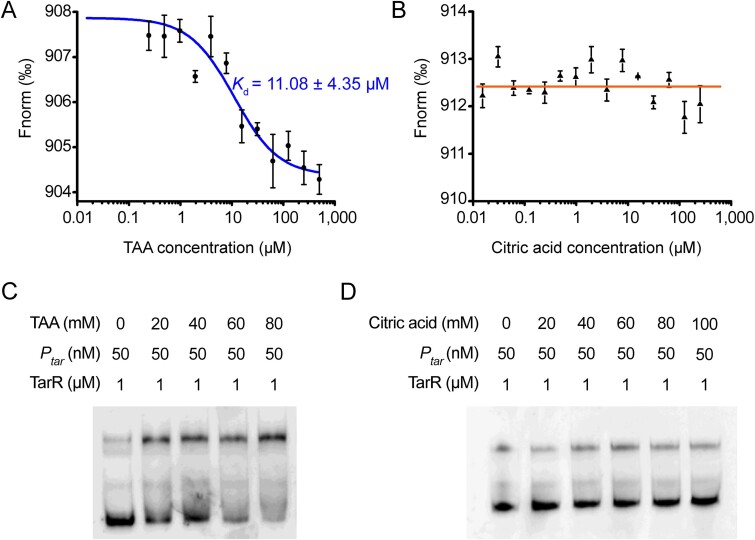
TAA directly binds to TarR protein. (A) MST analysis of the interaction between TarR and TAA, with citric acid (B) as a control. (C) EMSA tests the effect of TAA molecules on enhancing TarR-*P_tar_* interaction, with citric acid (D) as a control.

As mentioned before, the TarR protein belongs to the LysR transcriptional factor family, and its members usually regulate cellular functions by binding to certain small signaling molecules [30]. Furthermore, considering the bioinformatical prediction that the carboxyl domain of the TarR protein is responsible for substrate binding (Fig. 4A), and the potential regulation of TarR by TAA shown in qRT-PCR analyses (Fig. 4B and C), we propose that TAA is a signaling molecule that directly binds to TarR to activate the *tar* operon. Indeed, both MST (Fig. 5A) and thermal shifting (Fig. S6A) assays reveal a strong interaction between the TAA molecule and TarR protein (*K*_d_ = 11.08 ± 4.35 μM), whereas as a negative control, citric acid (C_6_) displays no binding (Fig. 5B and Fig. S6A). The amino and carboxyl domains of TarR, TarR_N1–91_ and TarR_C59–286_, were further shown to be responsible for *P_tar_*-DNA binding (Fig. S6B) and TAA-substrate binding (Fig. S6C), respectively. We then further conducted an EMSA assay to test the effect of TAA ligands on the binding efficiency of TarR to *P_tar_* and found that formation of TarR-DNA complexes is significantly enhanced as the levels of added TAA increase (Fig. 5C and D), demonstrating that TAA can stimulate TarR-*P_tar_* interaction.

In summary, the above results illustrate that the TarR protein first binds to TAA signaling molecules and then binds to *P_tar_* to activate the expression of the *tar* operon.

### TAA assimilation system is widely present in bacteria

**Figure 6:**
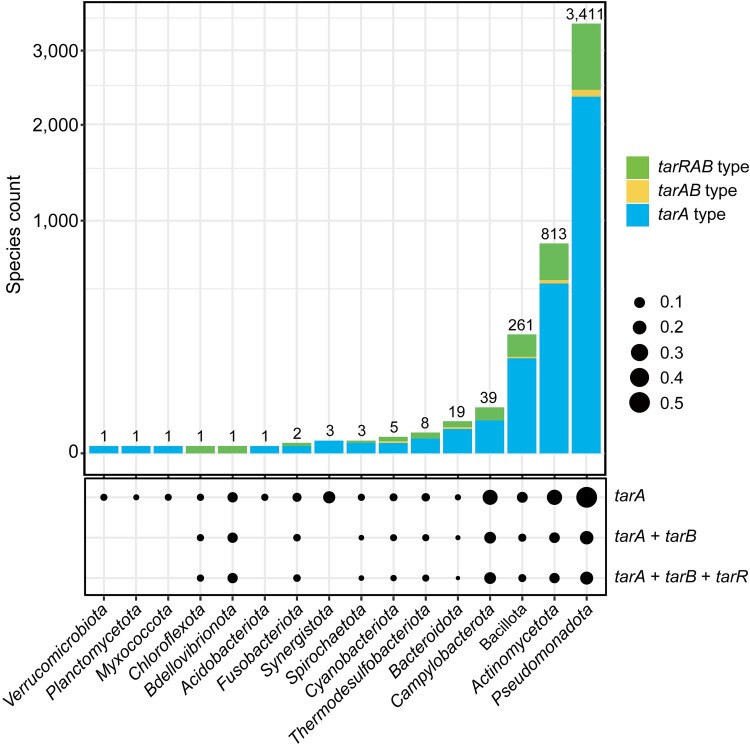
Phylogenetic distribution of TAA assimilation genes in bacteria. The numbers above each column are the total number of targets in the phylum, including *tarA-*type (containing only *tarA* homolog), *tarAB*-type (containing only “*tarA* + *tarB*” homolog), and *tarRAB*-type (containing “*tarR* + *tarA* + *tarB*” homolog) species. The dot plots show the relative abundances of TAA assimilation functions within each phylum.

To define the phylogenetic distribution of TAA-assimilation function in bacteria, we screened for the presence of *tarA*, *tarB*, and *tarR* homologous genes. Targets containing *tar* homolog were found to span across 16 bacterial phyla, 973 genera, and 4 570 species in total (Table S6 and Fig. 6). Of the 16 phyla, 11 contain *tarRAB*-type species that simultaneously harboring *tarA*, *tarB*, and *tarR* homologs (1 330 in total) (Table S6 and Fig. 6), indicating that these species have a complete assimilation system. Of the 11 phyla, *Pseudomonadota*, *Actinomycetota*, *Bacillota*, *Campylobacterota*, *Bacteroidota*, *Thermodesulfobacteriota*, *Cyanobacteriota*, and *Spirochaetota* are the top eight phyla with the largest number of target species among all the 16 phyla (Fig. 6). Meanwhile, we noticed that among *Pseudomonadota*, *Actinomycetota*, *Bacillota*, *Bacteroidota*, and *Cyanobacteriota* phyla, 142 species are of *tarAB*-type containing only (*tarA* + *tarB*) homologs, namely, with complete isomerization and transport functional organization (Table S6 and Fig. 6). For the remaining five of the 16 phyla, the target species are not only few in number but also belong to the *tarA*-type containing only unique *tarA* homologs (Table S6 and Fig. 6). Despite lacking a transport element, these bacteria are still considered to have the potential to use TAA. First, they encode the key element of the AI enzyme in the TAA assimilation process. Second, the lack of *tarB* homologs may be due to the high diversity of MFS sequences between and even within subfamilies [31–33]. There may be importers in alternative subfamilies with low similarity to TarB.

To further reveal the coverage of TAA assimilation ability within a phylum, the analysis of the ratio of target species within a phylum to total sequenced species of the phylum is also shown (Fig. 6). In the top 1 phylum *Pseudomonadota*, more than half of the species (3 411_target_/6 600_total_) encode AI (Tables S6 and S7), and more than 31.1% (1 060/3 411) further encode *tarB* or even *tarR* alternatives (Table S6), indicating that *Pseudomonadota* is a representative phylum that generally adopts TAA assimilation strategy; in *Actinomycetota* and *Campylobacterota*, the above ratios are 22.9% (813_target_/3 553_total_) and 34.4% (280/813), 22.2% (39_target_/176_total_) and 48.7% (19/39), respectively (Tables S6 and S7), which also indicate that TAA assimilation function is dominant in these phyla. Detailed information on the classification, homology type, and protein sequence identities of all bacterial targets are listed in Table S6.

### TAA-assimilating bacteria are also widely distributed in the natural environment

We collected 32 samples (Table S4) to explore the distribution of TAA assimilation bacteria in the natural environments and found that many TAA-utilizing bacteria could be isolated from these samples, yielding a total of 77 strains, and 66 of them were identified (Fig. S7A). These bacteria belong to three phyla, namely *Pseudomonadota*, *Bacillota*, and *Actinomycetota*, involving 12 genera and 27 species, which successfully verified the widespread nature of TAA assimilation species in bacteria taxa. These isolates are particularly concentrated in the phylum *Pseudomonadota* (46/66, 69.7%), which ranks first in the phylogenetic results (Fig. 6) and is consistent with the early reports that identified many *Pseudomonas* spp. using TAA molecules [23]. However, our isolation results differ from that report, which indicated that Gram-positive bacteria (involving only two phyla of *Bacillota* and *Actinomycetota*) rarely use TAA. In our work, we found that 29.6% (8/27) of the isolated species are Gram-positive, which may reflect that uptake of TAA carbon sources by Gram-positive bacteria in nature is real. Subsequently, the genomes of 12 representatives of the 66 strains were analyzed by tblastn. These strains differ in species, living environments, and lifestyles, including plant- [34] and animal- pathogenic [35–39], plant-beneficial [40–44], and free-living [45]. Except for strain 56 (*Pantoea agglomerans* sp.) and strain 60 (*Serratia marcescens* sp.), the two species newly identified in this work that were able to use TAA but did not have TarA-homologs, the remaining 10 strains were found to have at least one homologous sequence (Table S4). In total, we obtained 16 candidate sequences from 10 strains (Table S4) and found that 70% of the strains (7/10; strain 7, 16, 23, 32, 41, 68, and 69) encode active AI enzymes, and two of them, AI-7-1 (^**^P = .008) and AI-16-1 (^**^P = .004) in strain 7 and strain 16, exhibit significantly higher catalytic efficiency than TarA of FZB42 (Fig. S7C). In comparison, the forward or reverse isomerization activities of AI-4 (3.5%, 8.6%), AI-22 (8.2%, 24.7%), and AI-79 (0.7%, 0.4%) proteins are quite low (Table S4), although strains 4 (*Aeromonas veronii* sp.), strain 22 (*Acinetobacter pittii* sp.), and strain 79 (*Providencia rettgeri* sp.) encoding these genes grew well on the ACO plate (Fig. S7A), implying that weak enzymatic performance may be sufficient for survival in nature, or these AIs may require special biochemical conditions for catalysis, or these bacteria may encode unique and unknown AI sequence(s).

Together, these results suggest that TAA assimilation was active and prevalent in bacterial carbon utilization and underscore the importance of TAA as a natural carbon nutrient.

### Growth advantage of TAA-assimilating *B. velezensis* strain in soil

**Figure 7:**
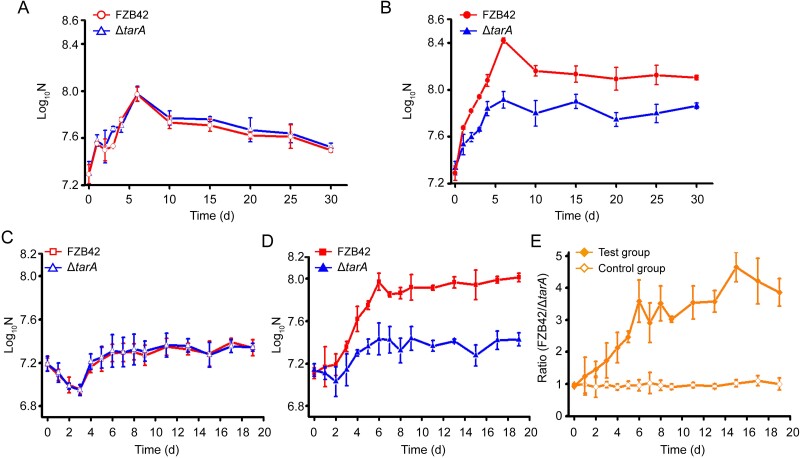
Individual and competitive colonization of the TAA assimilator FZB42 and its mutant Δ*tarA* disabled in TAA assimilation in sterile soils with and without TAA addition. (A) and (B), individual colonization in sterile soils without and with TAA addition, respectively. (C) and (D) competitive colonization in sterile soils without and with TAA addition, respectively. (E) Ratio of the number of FZB42 cells to the number of Δ*tarA* cells counted in (D).

Based on experimental results, we deduced that the function of the TAA assimilation system is to confer a survival advantage to assimilators in natural environments where TAA is present. To test this hypothesis, we examined the growth rate and final populations of the TAA-assimilating strain FZB42 and the non-assimilating strain Δ*tarA* in sterile soil (naturally TAA-absent, Fig. S8) without other competing microbiota. This approach allowed us to focus solely on the effects of TAA assimilation ability on competing bacteria. When examined individually in soils without TAA addition, the two strains show indistinguishable growth rates and similar final population levels over the course of the tests (30 days) (Fig. 7A). However, when TAA was added and present in soils, the TAA assimilation strain FZB42 reproducibly grew to 2–3 times the size of the non-assimilation strain (Fig. 7B), suggesting that TAA-assimilating strain has a considerable survival advantage in the presence of TAA.

### Competition between TAA-assimilating and non-assimilating *B. velezensis* strains in soil

To further determine the competitive advantage conferred by the TAA assimilation system in FZB42 when competing with Δ*tarA* during survival in soil, cultures of both strains were mixed at a population ratio of 1:1 and inoculated into sterile soils with or without TAA. In the absence of TAA, neither strain becomes dominant (Fig. 7C), and the distribution of strains in the total bacterial population remains ~1:1 throughout the experiment (Fig. 7E). However, when TAA was added in the soils, strains utilizing TAA became the dominant members of the test group (Fig. 7D), as the ratio favors the FZB42 assimilator (from 0.94 to 3.86 during 0–19 days) (Fig. 7E). This confirms that bacteria equipped with a TAA-assimilation system have a clearly competitive advantage in environments where TAA is present.

## Discussion

**Figure 8:**
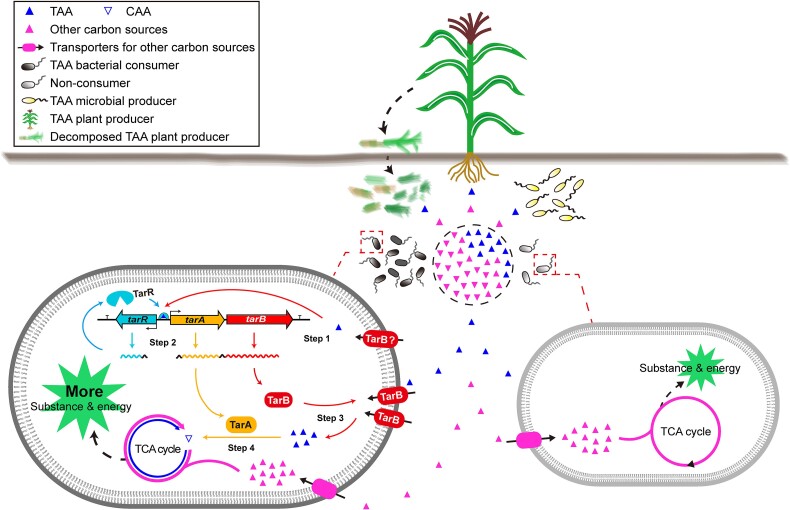
Proposed models for how TAA assimilation confers survival advantage on bacteria in natural environment. TAA metabolites can be secreted by bacteria (such as *B. thuringiensis* and *Pseudomonas* spp.) or by the roots of plants (such as maize, grass, and sugar cane) as a carbon source. Returning the plant tissues to the field can also increase TAA content. When TAA is present, both bacterial consumers (e.g. *B. velezensis*) and non-consumers that do not encode AI enzymes will use the most common carbon sources (e.g. glucose and citric acid), but only the former is able to use TAA. Therefore, in the same living space, TAA assimilation can give consumer bacteria more nutrients, thereby achieving more vigorous growth and larger population size than non-consumer bacreria, and eventually winning the survival competition. Specifically, the molecular mechanism by which bacteria absorbing environmental TAA as a carbon source is as follows*.* Step 1. Perception. The detection of TAA molecules may be achieved by leaky expression of TarB protein, as evidenced by the detectable *tarB* transcripts by RT-PCR in the absence of TAA (see Fig. S11). Step 2. Activation. In the cytoplasm, the imported TAA acts as a signal to bind directly to the TarR protein, a positive specific regulator of TAA assimilation function. TarR then directly binds to *P*_*tar*_ to significantly activate tar expression and the production of TarA and TarB proteins. Step 3. Transport. TarB importers robustly transport available environmental TAA into the cell. Step 4. Isomerization. TarA enzymes serve as an AI to convert TAA into CAA, incorporating the carbon source into the central metabolism of the TCA cycle, and promoting the production of more building materials and energy.

Here, by studying the model strain *B. velezensis* FZB42 for microbe-environment interaction, we identified the bacterial genetic determinants of *tarR* and the *tar* operon responsible for activation of TAA assimilation and TAA sensing, import, and isomerization, confirming the growth and competitive survival advantages provided by this assimilation system (Fig. 8), as well as its widespread distribution in the bacterial domain and in a variety of environmental bacteria.

The *tar* operon is a new member of the bacterial inducible operon group. It shows a similar regulatory pattern to some classical operons (such as the *lac* for lactose catabolism [46], *rha* for rhamnose catabolism [47], and *ttd* for tartrate catabolism [48]), whose expression targets a substrate molecule for degradation and requires the molecule itself to first serve as an inducer for activation. Thus, TAA molecule, TarR protein, and the *P_tar_* promoter may provide new regulatory alternatives for synthetic biology applications.

To our knowledge, all bacterial AIs identified to date exhibit the same enzymatic properties *in vitro*, namely, interconversion between CAA and TAA, and more uniquely, a preference for TAA formation [9, 49]. However, once inside the cell, these AIs “differentiated” in functions to specifically mediate TAA consumption (e.g. TarA) or biosynthesis (e.g. TbrA). Comparison of the *in vitro* enzymatic data for TarA and TbrA reveals similar and classic AI catalytic behavior (Table S5) [50]. By expressing *B. thuringiensis tbrA* in *B. velezensis* and *B. velezensis tarA* in *B. thuringiensis*, we found that TarA and TbrA both belong to the PrpF superfamily and can functionally substitute for each other (Fig. S9), suggesting that there are other factors besides AI enzymes that determine the assimilation or production of TAA. TAA transporters are one of the most likely determinants. In bacteria, TAA transporters may be highly differentiated in the transport directions of TAA, which determines the source and function of TAA molecules, which are nutrients imported from the environment or toxin synthesized for export. As in the nematode pathogen *B. thuringiensis*, once the AI enzyme in the cytoplasm isomerizes CAA to TAA, the exporter protein TbrB immediately pumps TAA out of the cell to alleviate its cumulative toxicity to the TCA cycle, thereby continuously driving the isomerization continuously toward and forming TAA nematicides [9]. In *B. velezensis*, the delivery of TAA from the environment into the cell by the TarB importer (Fig. 8) may lead to a rapid increase of TAA level in the cytoplasm. Although AI favors CAA substrates, high concentrations of TAA substrates would repress TAA formation and shift AI equilibrium to TAA consumption.

AI enzyme holds promise for industrial production of TAA via biosynthesis. Novel AI sequences distinct from TarA have the potential to be isolated from bacterial species clustering in Classes I, II, or III in the AI evolutionary analysis (Fig. S10), or simply by ACO plate selection. Directed evolution of AI enzymes with desirable properties is also expected to accelerate the future applications of TAA in more challenging fields such as medicine and agriculture.

The identification of the TarB importer following the discovery of the *B. thuringiensis* exporter TbrB further underscores the important role of orientation-specific transporters in mediating TAA-related physiology in bacteria. TarB and TbrB were found to have no sequence similarity and were classified into two independent subfamilies of 2.A.1 and 2.A.7, respectively, of the MFS (Transport Classification Database). These findings reflect the functional and sequence divergence of TAA transporters and indicate that highly independent groups of MFS or more complex subgroups that unidirectionally import or export TAA may have evolved in bacteria; in addition, bidirectional TAA transporters or non-specific transporters of TAA and its structural analogs may also exist. Therefore, the limitations of using the only available importer gene sequence, *tarB*, to predict TAA-assimilating bacteria containing importer homologs should be recognized. To solve these issues, the identification of more TAA transporters in bacteria is urgently needed.

As early as 1961, it was discovered that TAA metabolism is inducible in bacteria [20]. The identification of TarR has unraveled the regulatory mechanism, increased the regulatory scope of LTTRs, and deepened our understanding of microbial economic strategies for survival. Recent studies have shown that nutrients, in addition to being valuable sources of carbon and/or nitrogen, also act as signaling molecules in nutrient-assimilating bacteria to express genes that promote survival [51]. Similarly, in the TAA-TarR signaling pathway of the important plant growth-promoting rhizobacterium (PGPR) *B. velezensis* FZB42, we further found 90 TarR potential regulon genes whose promoter regions contained at least two key recognition motifs “TTATAA” as the *P_tar_*, implying that TarR or TAA may regulate more bacterial functions required for survival in plant-associated environments. Characterization of the genes and functions is essential to understand the story of nutrition initiation.

The widespread distribution of TAA assimilation systems in the bacterial domain reflects that TAA is a common and important component of the carbon source pool in nature. In addition to free-living or plant-associated environments, TAA molecule was also detected in animals [52]. In this study, we found that many animal-endophytical bacteria were predicted to be TAA-assimilating bacteria (Table S6), such as *Acidaminococcus intestine* and *Fusobacterium mortiferum*, *Peptoniphilus ovalis*, *Desulfovibrio porci*, *Cloacibacillus porcorum*, *Sporomusa termitida* and *Intestinirhabdus alba*, and *Shewanella marinintestina*, which have been reported to colonize the intestines of humans [53], monkeys [54], pigs [55], rabbits [56], insects [57, 58], and marine fish [59], respectively. Can TAA nutrition influence the growth and colonization of assimilating bacteria in the animal host? Does TAA in the animal come from exogenous food intake or endogenous biosynthesis, and if the latter, what are the genes and pathways? These questions are unknown and valuable research topics.

Metabolic adaptation, especially to the carbon sources, is a critical determinant of bacterial growth, colonization, and ecological function in different environments [60, 61]; indeed, bacteria employ a variety of flexible strategies to achieve this. Bioinformatics evidence shows that genes encoding carbon metabolism categories have the strongest evolutionary conservation among all gene function categories in bacteria [60], supporting the idea that carbon metabolism is a fundamental and precondition in survival interactions. Experimental evidence from other plant-associated carbon source cases, such as opines [62], mimosine [63], and tartaric acid [64], also confirms that the greater and more diverse the carbon catabolism, the better the growth and colonization. Strengthening the link between carbon production and assimilation between the environments and microorganisms may help improve the performance of target microorganisms in the microbiota and shape the related structures. On this basis, beneficial microorganisms that are often unable to compete with the resident microbiota can be modified through synthetic biology to utilize specific nutrients, such as TAA (Fig. 8), to greatly improve the application potential for probiotics.

## Supplementary Material

Supplemental_material20241013_wrae198

TableS1_wrae198

TableS2_wrae198

TableS3_wrae198

TableS4_20241002_wrae198

TableS5_wrae198

TableS6_wrae198

TableS7_wrae198

## Data Availability

Genome data for 12 isolated TAA-assimilation bacteria was deposited in the NCBI BioProject database under the accession number of PRJNA1119364.
