# Supplementary material for: Trans-aconitic acid assimilation system as a widespread bacterial mechanism for environmental adaptation
Source: ISME J. 2024 Oct 7;18(1):wrae198. doi: 10.1093/ismejo/wrae198 (PMC11495376; doi:10.1093/ismejo/wrae198)
Supplement: Supplemental_material20241013_wrae198 [file supplemental_material20241013_wrae198.docx]

***Supplementary Material for***

***Trans*-aconitic acid assimilation system as a widespread bacterial mechanism for environmental adaptation**

Running title: *Trans*-aconitic acid use in bacteria.

Cao Zheng^a^, Dingqi Liu^b^, Xinyu Lu^c^, Huijun Wu^d^, Jingyi Hua^a^, Chuang Zhang^a^, Kang Liu^a^, Changchun Li^a^, Jin He^b*^ and Cuiying Du^a*^

^a^Hubei Province Research Center of Engineering Technology for Utilization of Botanical Functional Ingredients, Hubei Key Laboratory of Quality Control of Characteristic Fruits and Vegetables, College of Life Science and Technology, Hubei Engineering University, Xiaogan, Hubei 432000, China.

^b^National Key Laboratory of Agricultural Microbiology, Hubei Hongshan Laboratory, College of Life Science and Technology, Huazhong Agricultural University, Wuhan, Hubei 430070, China.

^c^State Key Laboratory of Biocatalysis and Enzyme Engineering, Hubei Hongshan Laboratory, Hubei Collaborative Innovation Center for Green Transformation of Bio-Resources, Hubei Key Laboratory of Industrial Biotechnology, School of Life Sciences, Hubei University, Wuhan, Hubei 430062, China.

^d^Key Laboratory of Integrated Management of Crop Diseases and Pests (Ministry of Education), College of Plant Protection, Nanjing Agricultural University, Nanjing 210095, Jiangsu, China.

^*^Corresponding authors: Cuiying Du, College of Life Science and Technology, Hubei Engineering University, No.272 Jiaotong Avenue, Xiaogan, Hubei 432000, China. Email: ducuiying.123@163.com; Jin He, College of Life Science and Technology, Huazhong Agricultural University, No.1 Shizishan Street, Hongshan District, Wuhan, Hubei 430070, China. Email: hejin@mail.hzau.edu.cn.

| **Page** | **Content** |
| --- | --- |
| **3** | **Fig. S1** The strong TAA assimilation ability of *B. velezensis* FZB42 and bioinformatics analysis of its potential TAA assimilation genes |
| **4** | **Fig. S2** Growth of *B. velezensis* FZB42 and Δ*tar* mutants in liquid LB (A) and ACO (B) media |
| **5** | **Fig. S3** Enzymatic characterization of optimal pH, ionic strength, metal ion effects, and kinetic parameters of TarA |
| **6** | **Fig. S4** Glucose residual levels in supernatants of FZB42 and Δ*tarB* strains cultured in modified ACO liquid medium |
| **7** | **Fig. S5** EMSA determination of the effect of the “TTATAA” palindromic sequence in mediating *P_tar_*-TarR interaction |
| **8** | **Fig. S6** *In vitro* assay of interactions between TAA or *P_tar_* DNA ligand with TarR and its truncated fragments. |
| **9** | **Fig. S7** Determination of the AI activity of 16 TarA-homologous proteins identified from 12 selected strains out of 66 identified bacterial isolates |
| **10** | **Fig. S8** LC-Q-TOF-MS showing the absence of TAA nutrient in soils used for *B. velezensis* growth and competition experiments |
| **11** | **Fig. S9** AI exchange experiments |
| **12** | **Fig. S10** Evolutionary analyses of predicted AI sequences derived from potential TAA-assimilating bacteria |
| **14** | **Fig. S11** RT-PCR analysis of the transcript levels of *tarA* and *tarB* mRNAs in *B. velezensis* strains of FZB42 and Δ*tarR* with (+) and without (-) TAA induction |
| **15** | **Titles of Supplementary Tables for separately uploaded excel files** |

**
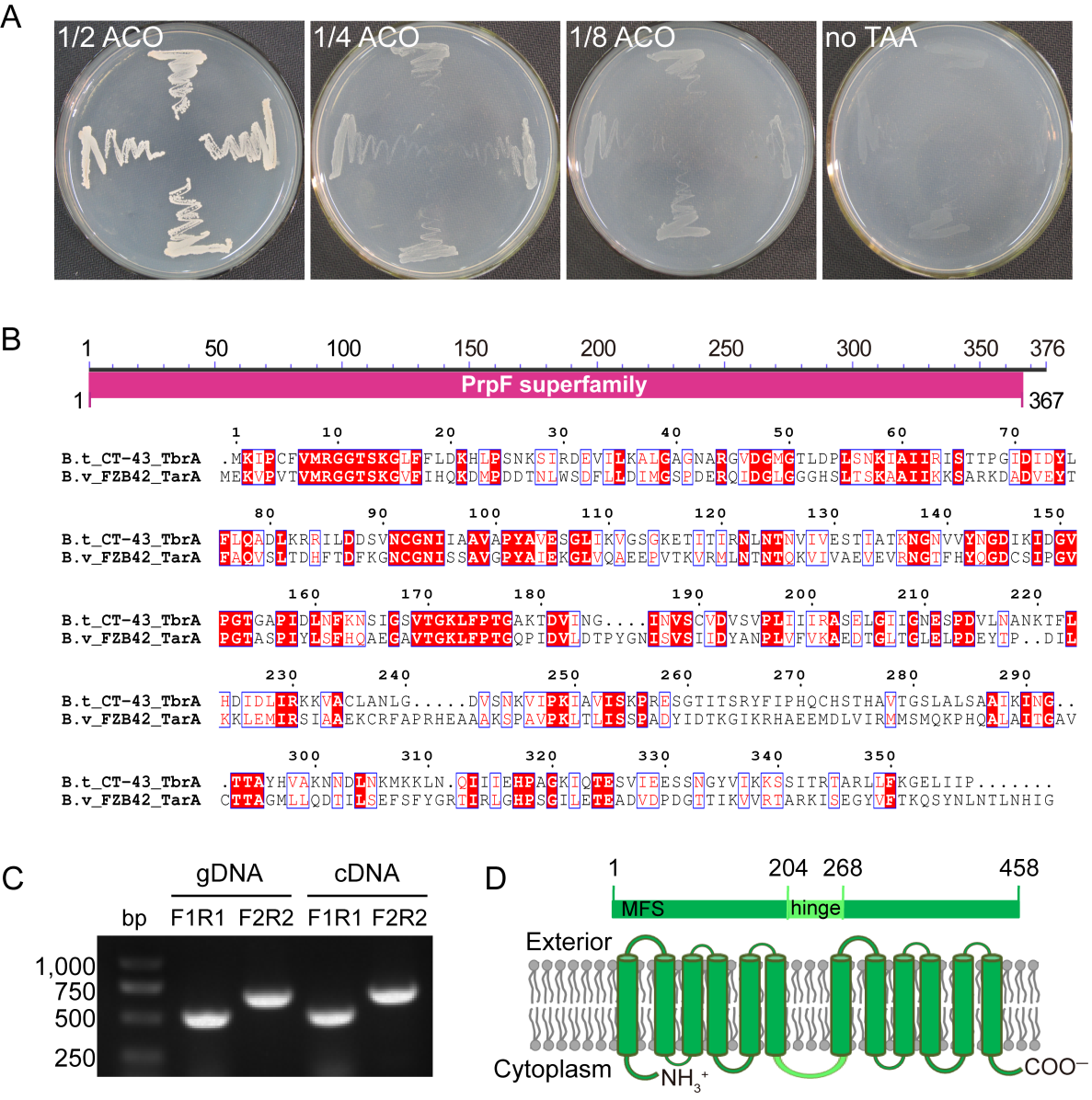
Fig. S1 The strong TAA assimilation ability of *B. velezensis* FZB42 and bioinformatics analysis of its potential TAA assimilation genes.** (A) Growth of *B. velezensis* FZB42 on modified ACO plates, with ACO medium formulated at one-half, one-fourth, and one-eighth the original TAA content or without TAA. (B) CD search of *B. velezensis* FZB42 TarA and sequence alignment with *B. thuringiensis* TbrA by ClustalX and ESPript. Identical residues are on a *red* background and residues with similar side chains are *boxed*. (C) Operon analysis of *tarA* and *tarB* genes in *B. velezensis* FZB42 by RT-PCR. gDNA: FZB42 genomic DNA. F1R1 and F2R2: DNA fragments within the *tarA* ORF, spanning the intergenic region between *tarA* and *tarB*, respectively. (D) Schematic secondary structure of the putative transmembrane protein TarB that contains a major facilitator superfamily (MFS) domain (*dark green*). Both the amino and carboxyl termini of TarB are located in the cytoplasm and contain six transmembrane helices each. Between the two sets of helices is a 64-aa hinge region (204-268 aa, *light green*) located in the cytoplasm (Transporter Classification Database).

**
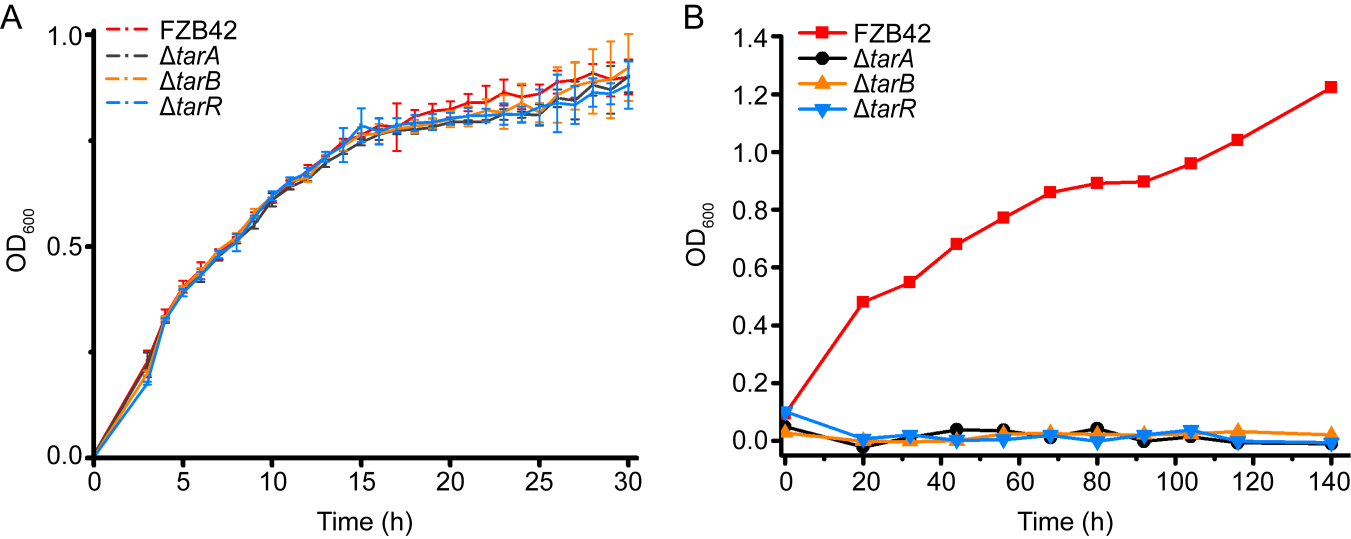
Fig. S2 Growth of *B. velezensis* FZB42 and Δ*tar* mutants in LB (A) and ACO (B) liquid media**. Data were measured using a microplate reader (A) and a spectrophotometer (B), respectively.

**
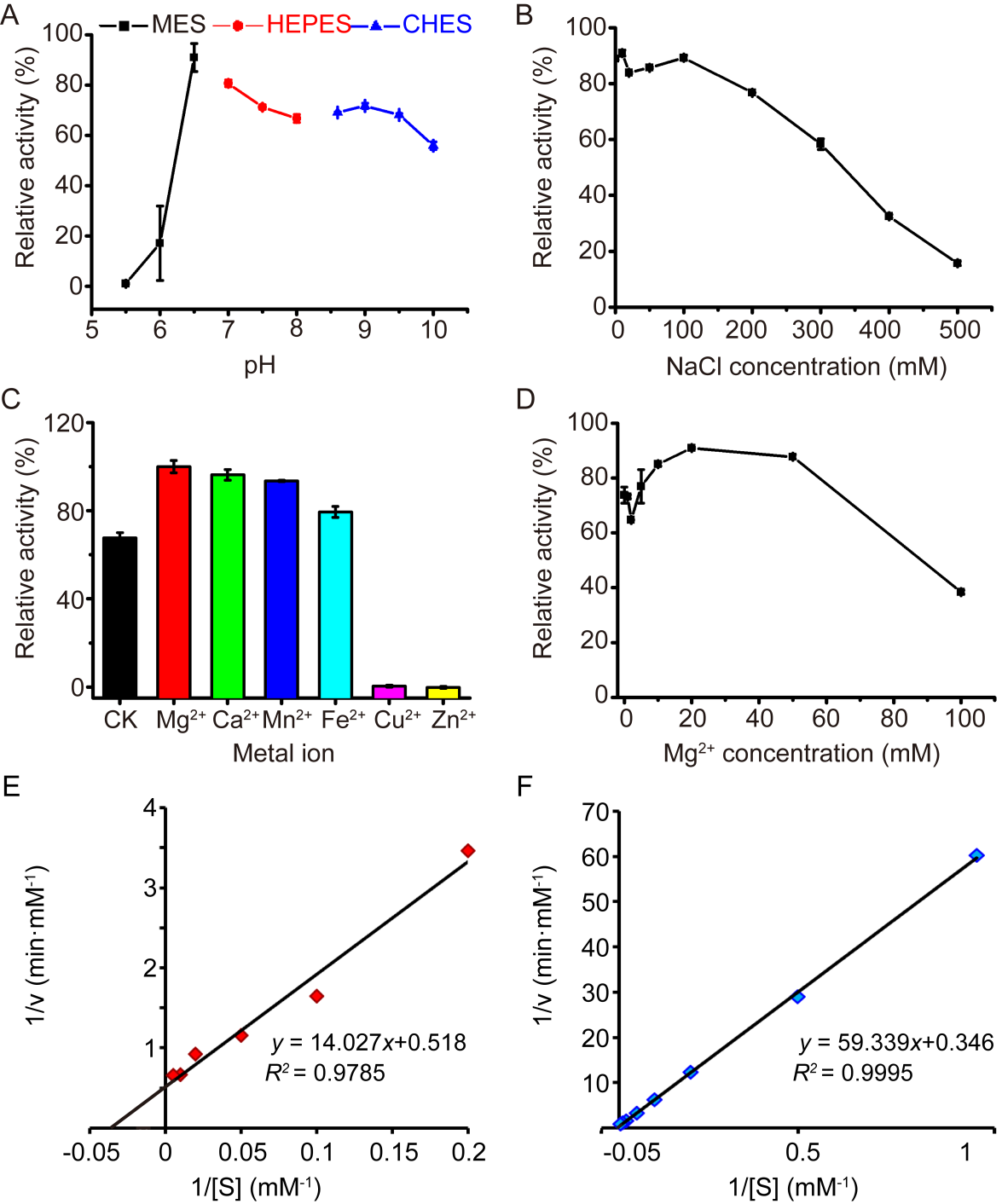
Fig. S3 Enzymatic characterization of optimal pH, ionic strength, metal ion effects, and kinetic parameters of TarA.** (A) Effect of pH on TarA activity under different pH conditions at 37°C. *Black* squares, 5.5-6.5 (100 mM MES); *red* circles, 7.0-8.0 (100 mM HEPES); *blue* triangles, 8.5-10.0 (100 mM CHES). (B) Effect of ionic strength on TarA activity under different final concentrations of NaCl ranging from 0 to 500 mM in 100 mM MES buffer (pH 6.5) at 37°C. (C) Effect of metal ions on TarA activity in 100 mM MES buffer (pH 6.5, 10 mM NaCl) containing various metal ions (MgCl_2_, CaCl_2_, MnCl_2_, FeCl_2_, CuCl_2_, and ZnCl_2_) at a final concentration of 10 mM at 37°C. (D) Optimal concentration of Mg^2+^ for TarA activity under different final concentrations of MgCl_2_ ranging from 0 to 100 mM in 100 mM MES buffer (pH 6.5, 10 mM NaCl) at 37°C. (E) Lineweaver-Burk plots of forward and (F) reverse reaction kinetic parameters of TarA.


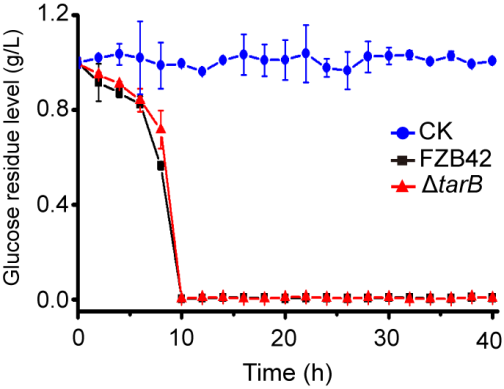
**Fig. S4 Glucose residual levels in supernatants of FZB42 and Δ*tarB* strains cultured in modified ACO liquid medium**. *Blue* circles represent no bacterium inoculation; *black* squares represent FZB42 strain inoculation; and *red* triangles represent Δ*tarB* strain inoculation.

**
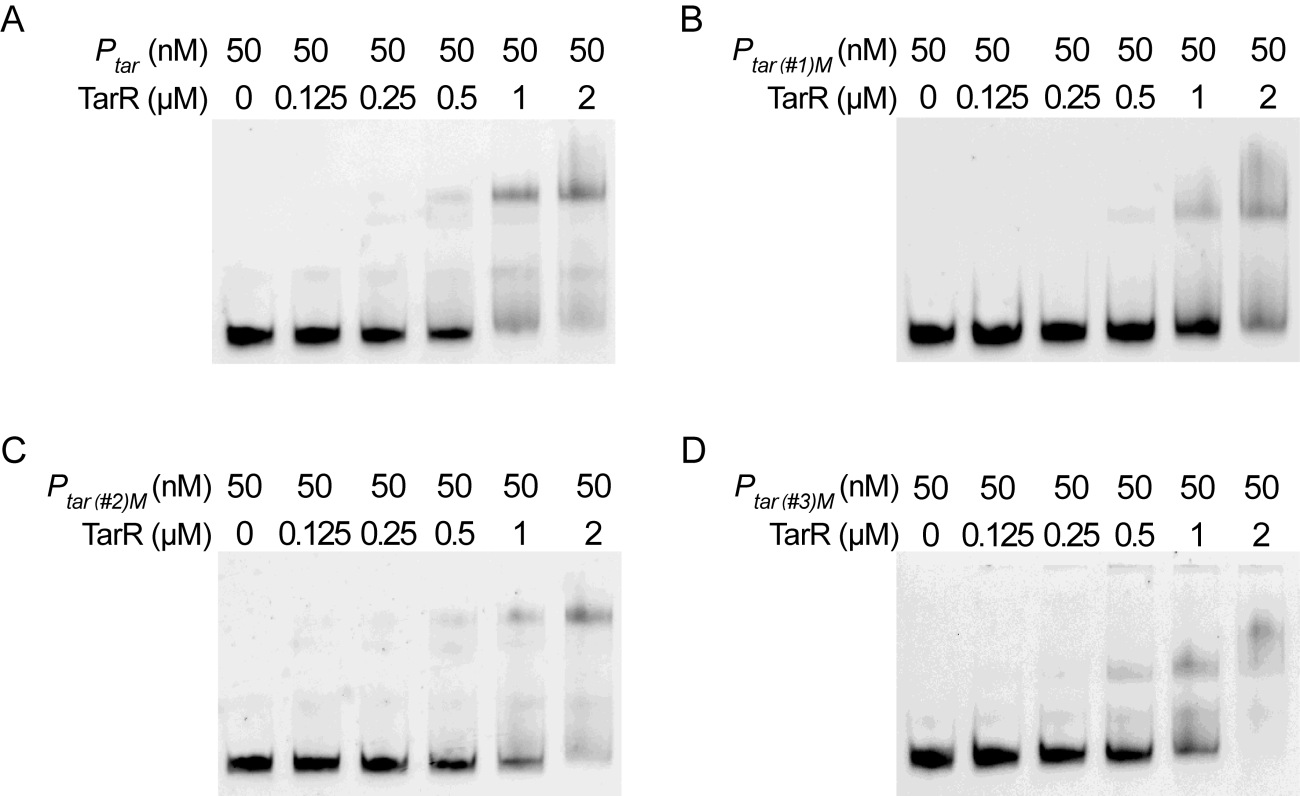
Fig. S5 EMSA determination of the effect of the “TTATAA” palindromic sequence in mediating *P_tar_*-TarR interaction.** (A) *In vitro* EMSA of TarR protein and *P_tar_* DNA with normal palindromic sequences of “TTATAA”, positive control. (B) EMSA of TarR protein and *P_tar_* DNA mutants, in which the #1, #2 (C), or #3 (D) palindromic motif of “TTATAA” is mutated to “CCGCGG”, as *P_tar(#1)M_*, *P_tar(#2)M_* , and *P_tar(#3)M_* , respectively.

**
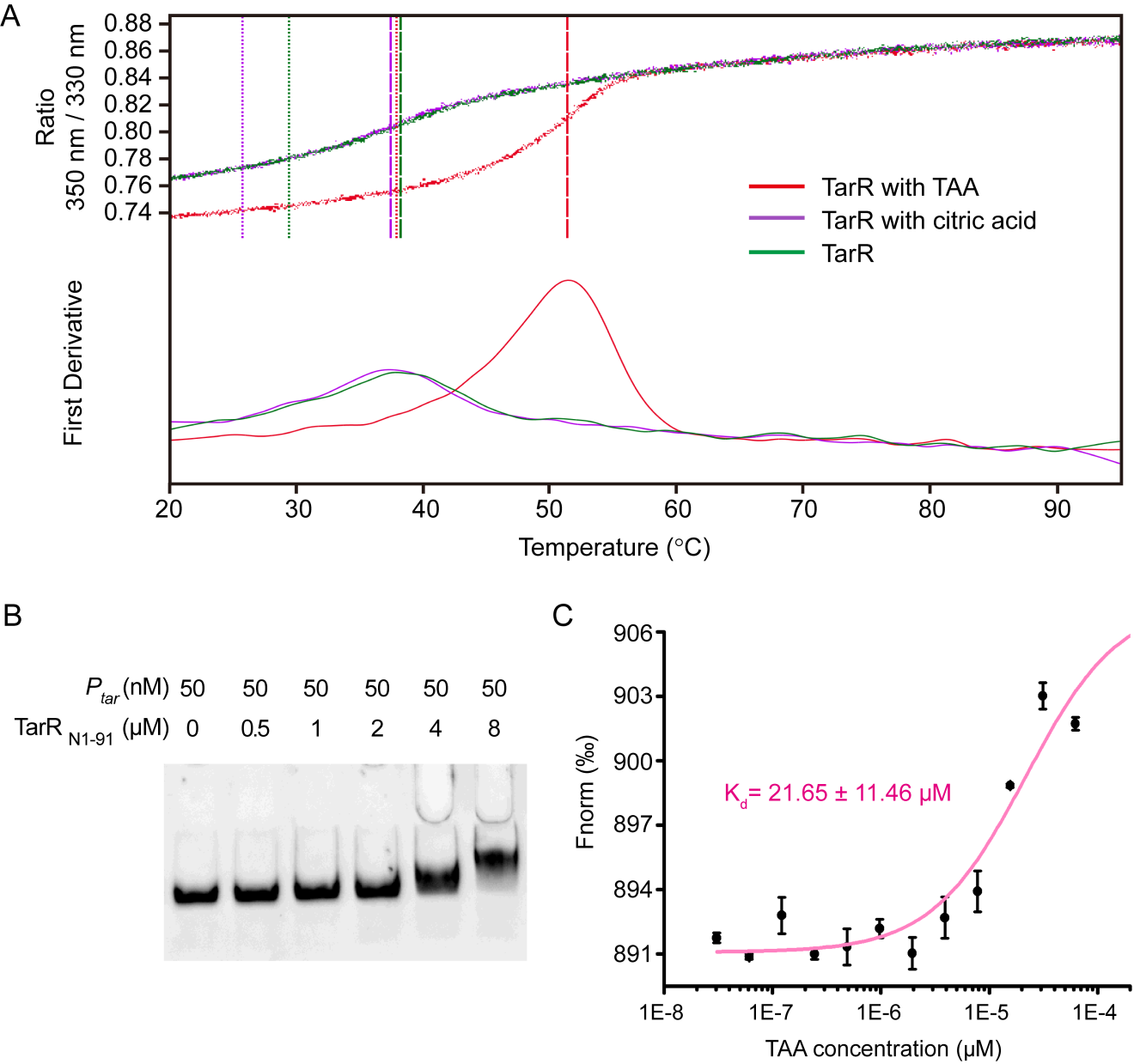
F****ig. S6 *In vitro* assay of interactions between TAA or *P_tar_* DNA ligand with TarR and its truncated fragments.** (A) Thermal-shift assay based on tryptophan fluorescence shows direct interaction between TarR and TAA. TAA (1 mM) and purified TarR protein (1 mg/mL) dissolved in 20 mM Tris-HCl (100 mM NaCl, pH 7.6) were mixed at a ratio of 1:9 and analyzed using Prometheus NT.48 (Nano Temper Technologies) with a temperature range of 20-95°C and a temperature slope of 1.0 °C/min, and PR.ThermolControl_2.1.5.7895 software. *T*_m_ was determined as the first derivative of the ratio of 350 nm to 330 nm fluorescence. Samples contain TarR protein alone (blank control, *green*), or TarR with citric acid (negative control, *purple*). (B) EMSA assay showing binding of TarR_N1-91_ and the amino terminal segment of TarR to the promoter region of *tar* operon. (C) MST assay showing binding between TarR_C59-286_, the carboxyl terminal segment of TarR, and TAA.

**
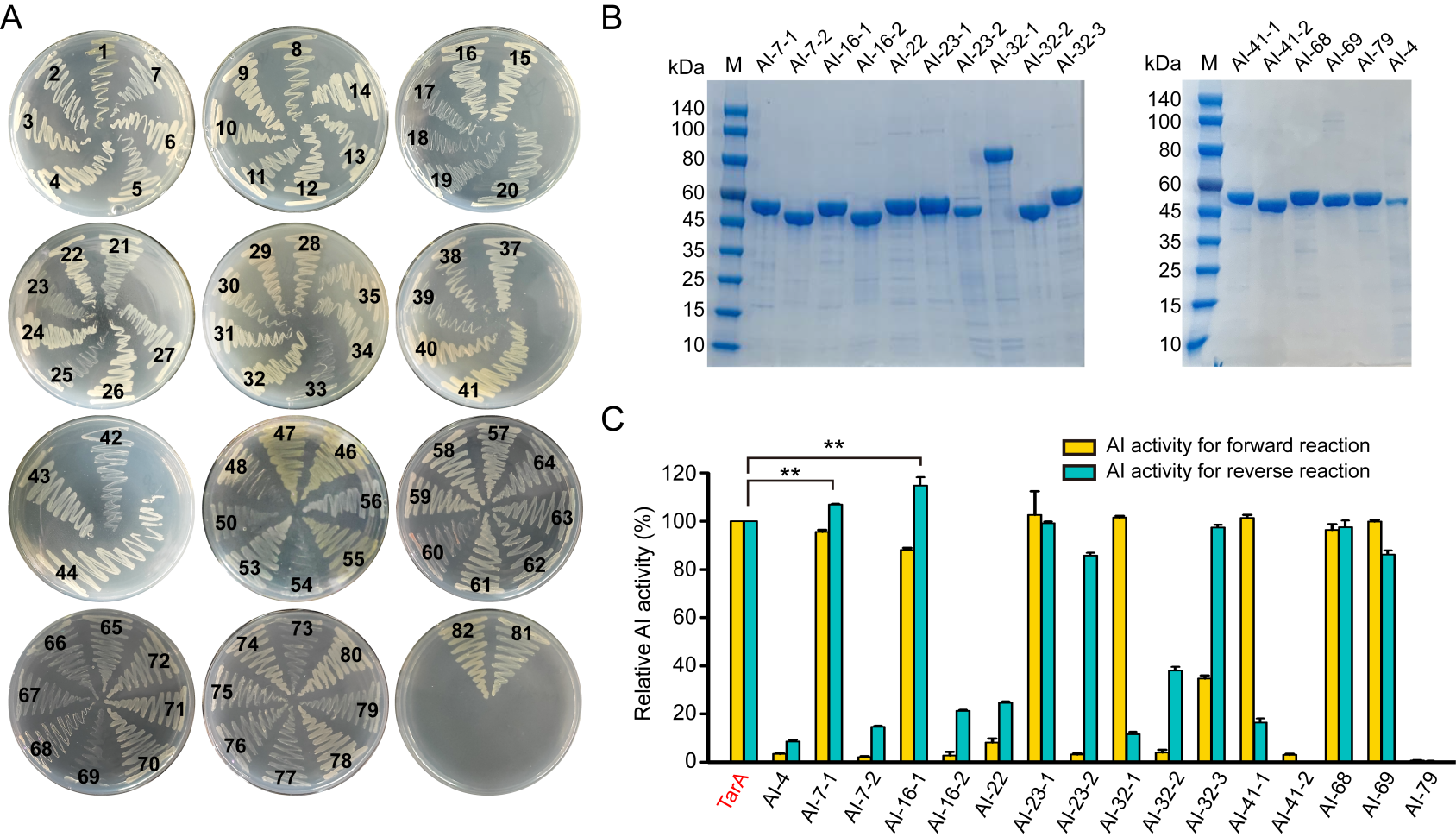
Fig. S7 Determination of the AI activity of 16 TarA-homologous proteins identified from 12 selected strains out of 66 identified bacterial isolates.** (A) Growth of 77 isolated TAA-assimilating bacterial strains on ACO medium plates. Photographs were taken after 24 h of culturing at 30°C. (B) Purification of 16 putative AI proteins derived from 12 representative bacterial strains isolated from natural environments. (C) Relative AI activities of 16 purified proteins. The 16 putative AI-encoding genes from each bacterial genome sequence were predicted by tblastn against the TarA protein sequence. By blastp analysis, the minimum coverage and identity of the 16 putative TarA AIs are 85% (AI-23-2) and 29% (AI-32-3), respectively (Table S7). The relative activity for the forward (*orange*) and reverse (*green*) reactions of each test AI were measured by adjusting and normalizing the final concentration of each enzyme to 1 μM, and calculated as the percentage ratios of the forward (or reverse) reaction activity of each test AI to the forward (or reverse) reaction activity of TarA.

**
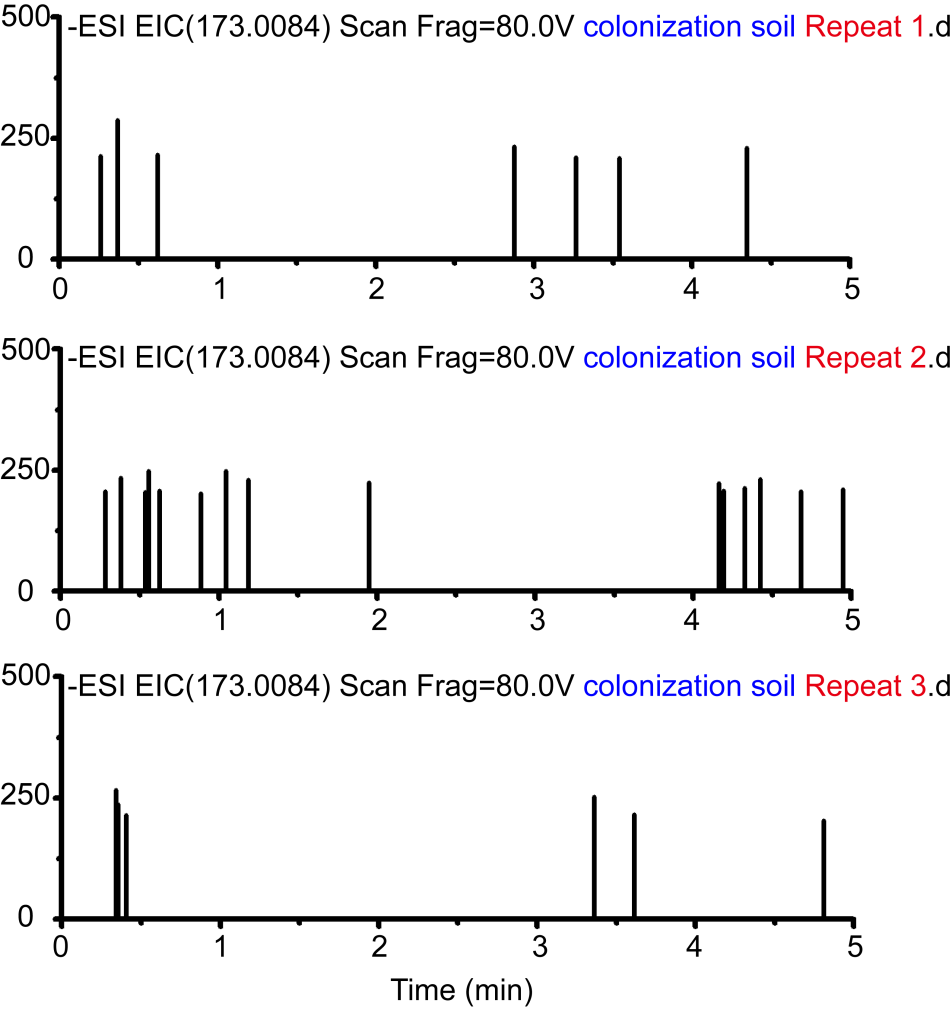
Fig. S8 LC-Q-TOF-MS showing the absence of TAA nutrient in soils used for *B. velezensis* growth and competition experiments.** Three independent soil samples collected from vegetable plots were analyzed as described in Materials and Methods.

**
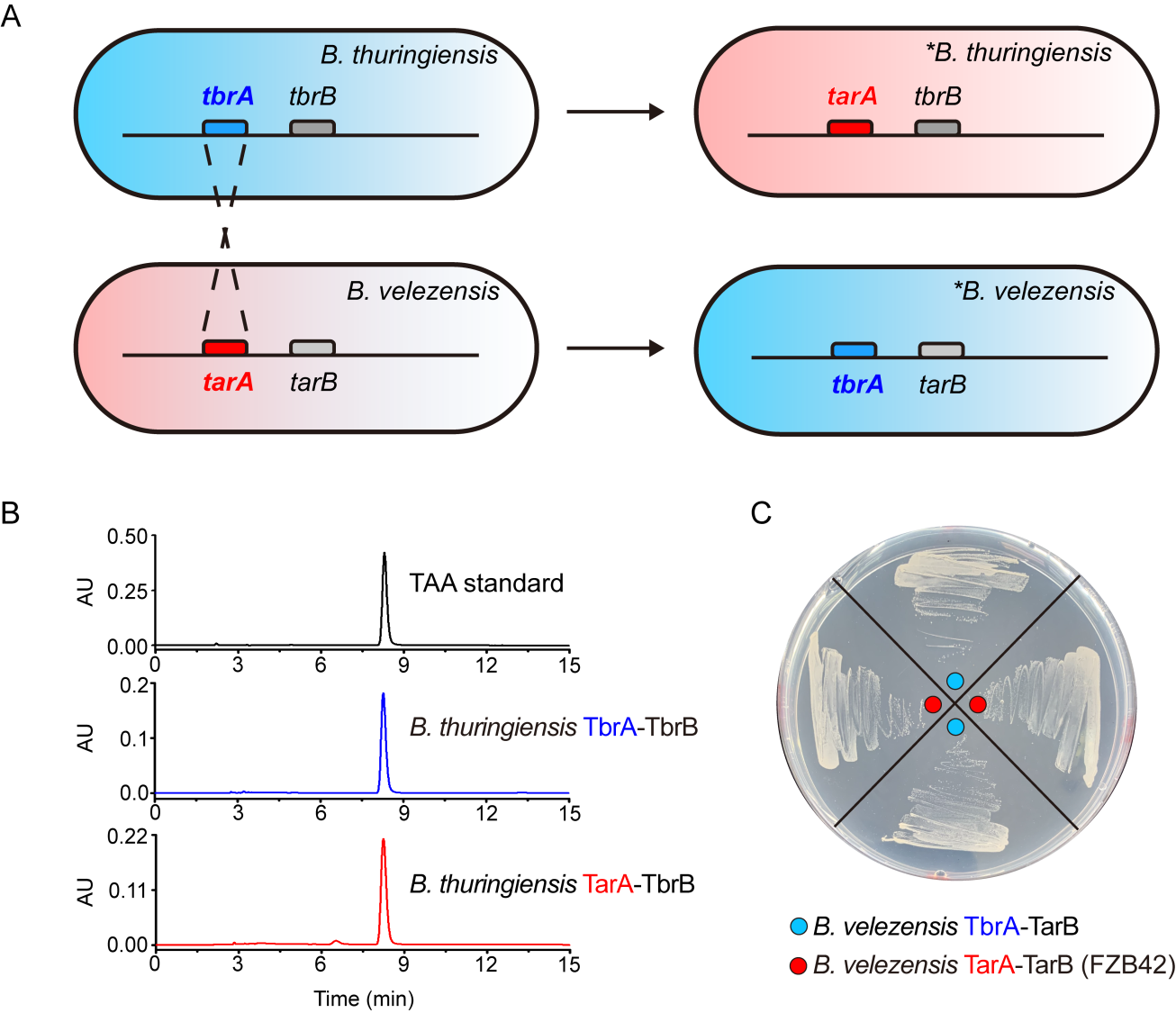
Fig. S9 AI exchange experiments.** (A) Design of TarA-TbrA exchange experiments in *B. thuringiensis* and *B. velezensis*. (B) HPLC analysis showing TAA production in recombinant *B. thuringiensis* TarA-TbrB. *B. thuringiensis* TbrA-TbrB was used as a positive control. (C) Growth assay on ACO medium plates showing the TAA-assimilation ability of *B. velezensis* TbrA-TarB. *B. velezensis* TarA-TarB (FZB42) was used as a positive control.


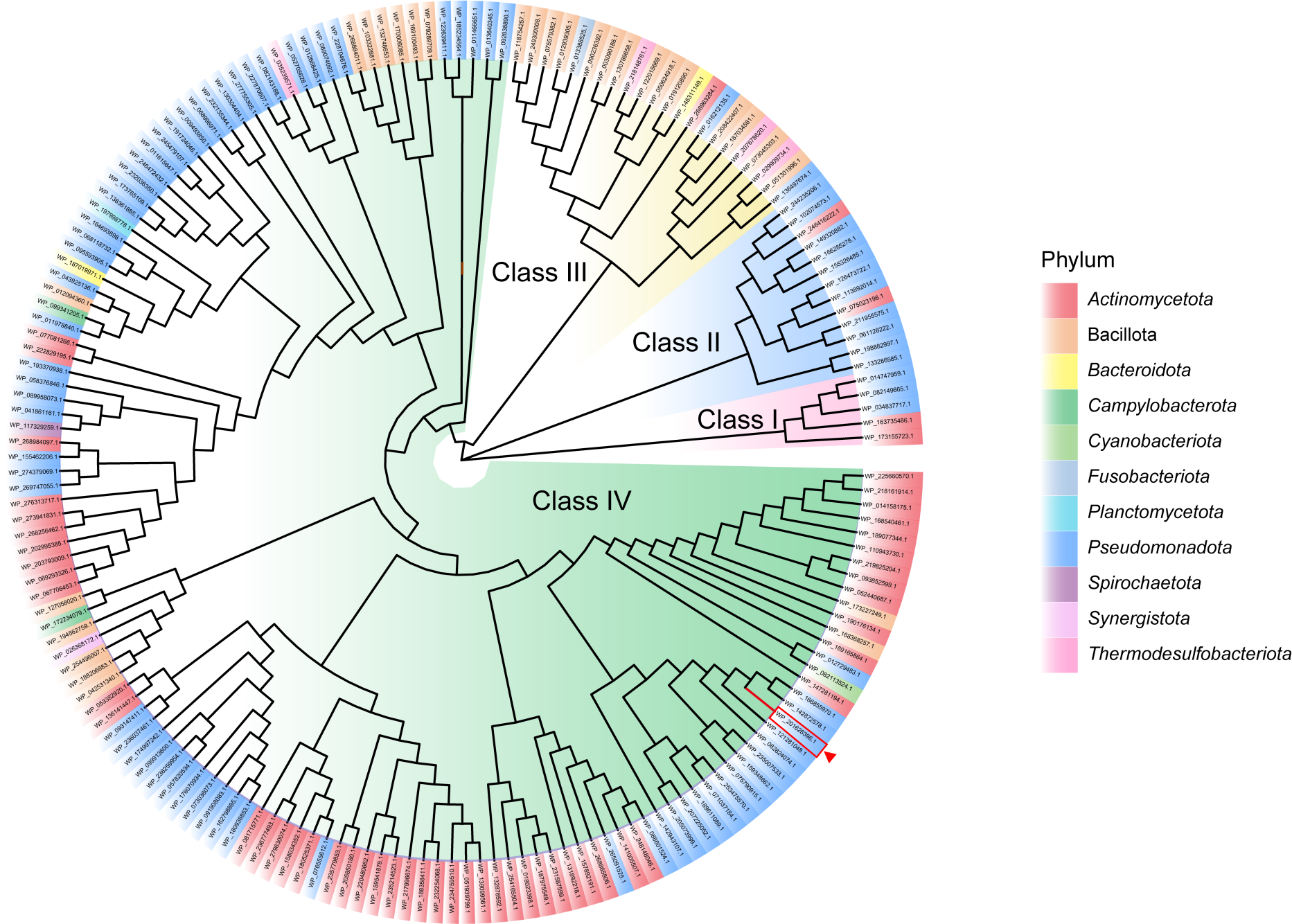


**Fig. S10 Evolutionary analyses of predicted AI sequences derived from potential TAA-assimilating bacteria.** All TarA homologous proteins were clustered at 50% sequence identity by CD-HIT (Version 4.8.1) and aligned by MAFFT. Maximum likelihood trees were constructed using Fasttree (Version 2.1.11) and further modified using the ggtree package (Version 3.8.2). The *red* triangle points to the protein cluster of WP_201628396.1, in which the *B. velezensis* TarA protein identified in this work is located.

**
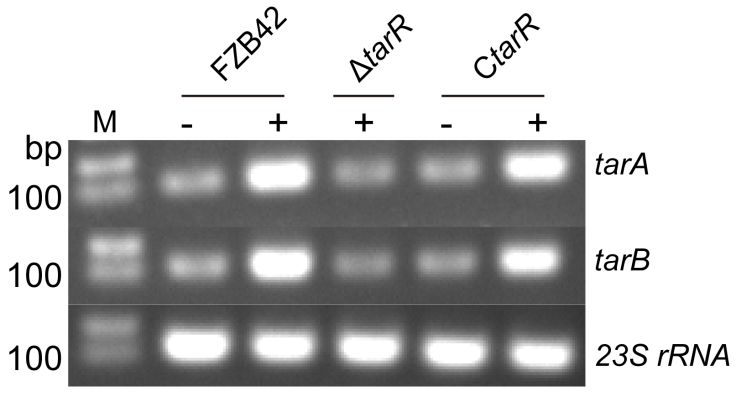
Fig. S11 RT-PCR analysis of the transcript levels of *tarA* and *tarB* mRNAs in *B. velezensis* strains of FZB42 and Δ*tarR* with (+) and without (-) TAA induction.**

**Titles of Supplementary Tables for separately uploaded excel files：**

**Table S1** Bacterial strains and plasmids used in this study.

**Table S2** Primers used in this study.

Restriction sites are underlined.

**Table S3.** The chemical properties of soil used for bacterial growth and competition experiments.

**Table S4** Summary information of the 32 environmental samples, 66 identified TAA-assimilating bacterial isolates, and 16 putative AI proteins.

* or ▲, indicate growth-promoting or pathogenic ecotypes respectively. Species in the “**Identified species**” column with *gray* background indicate selection of this species for AI expression and activity measurement. I, II, and III, indicate animal-associated, plant-associated and free-living styles, respectively, which were assessed according to published researches, and referenced. FR, forward reaction, RR, reverse reaction.

**Table S5** Kinetic parameters of *B. velezensis* TarA and *B. thuringiensi*s TbrA.

**Table S6** List of phylogeny distribution of TAA assimilation genes in bacteria.

**Table S7** The relative abundances of TAA assimilation function within each of the 16 target bacterial phylum.
